# Calcium lignosulfonate-induced modification of soil chemical properties improves physiological traits and grain quality of maize (*Zea mays*) under salinity stress

**DOI:** 10.3389/fpls.2024.1397552

**Published:** 2024-08-20

**Authors:** Yousef Alhaj Hamoud, Hiba Shaghaleh, Ke Zhang, Mohammad K. Okla, Ibrahim A. Alaraidh, Hamada AbdElgawad, Mohamed S. Sheteiwy

**Affiliations:** ^1^ National Key Laboratory of Water Disaster Prevention, Hohai University, Nanjing, China; ^2^ College of Hydrology and Water Resources, Hohai University, Nanjing, China; ^3^ The Key Lab of Integrated Regulation and Resource Development on Shallow Lakes, Ministry of Education, College of Environment, Hohai University, Nanjing, China; ^4^ Yangtze Institute for Conservation and Development, Hohai University, Nanjing, Jiangsu, China; ^5^ China Meteorological Administration Hydro-Meteorology Key Laboratory, Hohai University, Nanjing, Jiangsu, China; ^6^ Key Laboratory of Water Big Data Technology of Ministry of Water Resources, Hohai University, Nanjing, Jiangsu, China; ^7^ Key Laboratory of Hydrologic-Cycle and Hydrodynamic-System of Ministry of Water Resources, Hohai University, Nanjing, Jiangsu, China; ^8^ Botany and Microbiology Department, College of Science, King Saud University, Riyadh, Saudi Arabia; ^9^ Department of Botany and Microbiology, Faculty of Science, Beni Suef University, Beni-Suef, Egypt; ^10^ Department of Integrative Agriculture, College of Agriculture and Veterinary Medicine, United Arab Emirates University, Al Ain, Abu Dhabi, United Arab Emirates; ^11^ Department of Agronomy, Faculty of Agriculture, Mansoura University, Mansoura, Egypt

**Keywords:** calcium lignosulfonate, salinization, soil characteristics, grain quality, Zea mays

## Abstract

**Introduction:**

Salinity negatively affects maize productivity. However, calcium lignosulfonate (CLS) could improve soil properties and maize productivity.

**Methods:**

In this study, we evaluated the effects of CLS application on soil chemical properties, plant physiology and grain quality of maize under salinity stress. Thus, this experiment was conducted using three CLS application rates, CLS_0_, CLS_5_, and CLS_10_, corresponding to 0%, 5%, and 10% of soil mass, for three irrigation water salinity (WS) levels WS_0.5_, WS_2.5_, and WS_5.5_ corresponding to 0.5 and 2.5 and 5.5 dS/m, respectively.

**Results and discussion:**

Results show that the WS_0.5_ × CLS_10_ combination increased potassium (K 0.167 g/kg), and calcium (Ca, 0.39 g/kg) values while reducing the sodium (Na, 0.23 g/kg) content in soil. However, the treatment WS_5.5_ × CLS_0_ decreased K (0.120 g/kg), and Ca (0.15 g/kg) values while increasing Na (0.75 g/kg) content in soil. The root activity was larger in WS_0.5_ × CLS_10_ than in WS_5.5_ × CLS_0_, as the former combination enlarged K and Ca contents in the root while the latter decreased their values. The leaf glutamine synthetase (953.9 µmol/(g.h)) and nitrate reductase (40.39 µg/(g.h)) were higher in WS_0.5_ × CLS_10_ than in WS_5.5_ × CLS_0_ at 573.4 µmol/(g.h) and 20.76 µg/(g.h), leading to the improvement in cell progression cycle, as revealed by lower malonaldehyde level (6.57 µmol/g). The K and Ca contents in the leaf (881, 278 mg/plant), stem (1314, 731 mg/plant), and grains (1330, 1117 mg/plant) were greater in WS_0.5_ × CLS_10_ than in WS_5.5_ × CLS_0_ at (146, 21 mg/plant), (201, 159 mg/plant) and (206, 157 mg/plant), respectively. Therefore, the maize was more resistance to salt stress under the CLS_10_ level, as a 7.34% decline in yield was noticed when salinity surpassed the threshold value (5.96 dS/m). The protein (13.6 %) and starch (89.2 %) contents were greater in WS_0.5_ × CLS_10_ than in WS_5.5_ × CLS_0_ (6.1 %) and (67.0 %), respectively. This study reveals that CLS addition can alleviate the adverse impacts of salinity on soil quality and maize productivity. Thus, CLS application could be used as an effective soil amendment when irrigating with saline water for sustainable maize production.

## Introduction

Food security is threatened by salinization, water depletion, and environmental pollution. The total global water consumption in industrial, domestic, and agricultural activities is estimated to increase by 23% in 2025 and 40% by 2030 (Oumarou Abdoulaye et al., 2019; Boccaletti, 2023). Therefore, agriculture in most areas is estimated to face extreme food, water, and environmental crises over the next few decades (Kumar et al., 2022). Irrigation is the main water user, which is adopted to support agricultural production in regions where rainfall is insufficient. However, the shortage of water used for irrigation forced farmers to use low-quality water to irrigate crops (Dotaniya et al., 2023). Thus, everywhere saline irrigation is applied, the negative environmental impacts of salinization on agriculture are intensified (Mukhopadhyay et al., 2021; Sadak et al., 2024).

Salinization affects 33% of the total irrigated lands, and the amount of the world’s salt-affected soils is expected to increase yearly (Mukhopadhyay et al., 2021). This increase can be further hastened by the massive application of poor-quality water for irrigation, climate change, and excessive implementation of irrigation linked to poor drainage and exhaustive farming (do Nascimento et al., 2024). Under salt stress, crops must mainly cope with ionic and osmotic stresses. The osmotic stress is caused by reduced soil moisture potential, whereas the large salt captivation of crops causes ionic stress (Ludwiczak et al., 2021). Therefore, salinization restricts the acquisition of plant nutrients, initiates nutritious disorders, and eventually leads to crop yield losses (Alfadil et al., 2021; Ahmed et al., 2024). However, salt-resistant plants reduce sodium uptake in roots and shoots (Rady et al., 2011; Wang et al., 2022), as sodium accumulation in plant tissues is the main reason for crop deterioration under salt stress (Zhao et al., 2021; Atta et al., 2023). Therefore, the crop’s capability to eliminate the harmful sodium cations is one of the utmost tactics for reducing salinization stress.

Numerous strategies have been employed to decrease salinization effects on crops by submitting different organic materials, such as biochar, to soils (Mishra et al., 2023). Biochar is a carbonic matter generated by biomass thermal degeneration under high temperatures and low oxygen conditions (Zou et al., 2022). Biochar is of great interest due to its capability to improve soil carbon sequestration (Li et al., 2021b). Biochar can also improve the fertility of saline soils (Tang et al., 2023). Moreover, biochar enhanced soil chemical characteristics regarding soil pH, soil surface area, and soil cation exchange capacity under abiotic stresses (Yuan et al., 2023). However, applying biochar may limit crop yield by soil nutrients, causing a deficiency of nutrients available to crops (Hossain et al., 2020). Also, biochar’s frequent and long-term application causes soil compaction and degradation (Brtnicky et al., 2021). Moreover, some biochar is a potential source of soil pollution, although heavy metals are included in the biochar itself (Narayanan and Ma, 2022).

Alternatively, calcium lignosulfonate (CLS) application has great potential to regulate soil pH and enhance the fertility and quality of saline soils (Abbas et al., 2022). Calcium lignosulfonate is an amorphous material obtained during the sulfite pulping of softwood, biocompatible in soils with low cost (Liu et al., 2020). CLS is a complex organic polymer produced through lignin solubilization under alkaline conditions (Ghavidel Darestani et al., 2018; Liu et al., 2023). After completion of the pulping, the water-soluble calcium randomly lignosulfonate polymeric framework with three aromatic alcohols is separated from the cellulose, purified, acidified, evaporated, and spray dried. CLS addition has an obvious influence on the growth of plant roots, stimulates the improvement of chlorophyll, amino acids, and sugars in plants and aid in photosynthesis. Moreover, it has positively impacted shoot and root development, plant pigments, nourishing efficiency, and crop yield (Kok et al., 2021). CLS controls the stomata close and open on the leaves, thus increasing the plants’ ability under stressed conditions (Schulze et al., 2021). CLS application also improved soil health, plant–soil interactions, and rhizosphere microorganisms; moreover, the positive impacts of CLS application on crop root development were obvious (Elsawy et al., 2022). Furthermore, CLS could promote overall plant growth in maize as it has negative charges with a short-chain structure, simplifying the reaction with the salts (Ertani et al., 2019). Therefore, CLS addition could motivate root and shoot development, thereby boosting leaf growth, chlorophyll content, and crop yield (Kok et al., 2021). However, very limited information is available on the interactions between crop-soil-CLS particles and their relations to productivity and grain quality of maize with the addition of increasing CLS amounts under saline irrigation. Moreover, the physiological processes of plants and the observation of shoot development adaption during different stages are largely scarce. Therefore, soil is treated with CLS to adjust the soil properties, thereby enhancing maize productivity and quality under salinity stress is of utmost interest.

Globally, maize is the third leading food crop after rice and wheat. Salinity adversely impacts plant growth and physiology, leading to significant crop yield loss. Root–soil interactions regulate the plant growth level, and superior root development supports higher maize production (Gao et al., 2020). The maize–soil interactions are also varied by environmental variations and controlled by the availability of nutrients in the soil. Moreover, the interactions between roots and organic elements can affect overall maize physiological and biochemical attributes under salinity stress (Helaoui et al., 2023). However, the crop’s physiological attributes, nutrient uptake, and their relationships to the grain quality of maize with CLS application under salinity stress conditions are poorly understood. Therefore, the observation of maize responses to increasing rates of CLS application under salinity stress is vital to ascertain the accurate soil amendments to enhance maize productivity, and interpretations of the plant’s physiological attributes at different growth phases are essential for this objective. In addition, the water shortage has become a major constrain of agriculture production (Ingrao et al., 2023; Bakry et al., 2024). Therefore, soil quality should be enhanced by applying precise soil management tactics emphasizing irrigation approaches in crop production (Hamoud et al., 2022). To date, limited information on the effects of increasing CLS application on soil quality and maize productivity under salinity stress has been provided. Thus, to address the existing gap in knowledge, the present study hypothesized that CLS application could improve the soil chemical properties under salinity stress, which motivates the root physiological traits of maize. The study also assumed that the CLS addition could increase the availability of nutrients in the soil, increasing shoot physiological traits, nutrient uptake, and maize quality under salinity stress. To test this hypothesis mentioned above, this study evaluated the effects of CLS addition on soil chemical properties as well as the availability of nutrients in the treated soil under different levels of salinity stress. This study also determined the impact of CLS addition on the maize’s physiological performance and grain quality under varied levels of salinity stress. The guiding of this study would be of great development of sustainable agriculture, providing practical support and a theoretical base for precision soil and water management of maize. This study also identifies the mechanism by which CLS addition would improve soil quality and increase growth and yield of maize, elucidating the synergistic interaction between saline irrigation and CLS addition on grain yield and quality of maize.

## Materials and methods

### Study site description and soil properties

This experiment was carried out from June to November 2022 at the agricultural farm of Hohai University, Nanjing (31°57′N, 118°50′E), China. The weather of this zone is categorized as humid subtropical with four seasons. The annual mean temperature is 15.9°C, the maximum and the minimum temperatures are 43°C and −16.9°C, and the mean annual rainfall is 1,062 mm (Yang et al., 2018). The soil was collected from the topsoil layer (0 cm–15 cm), dried by sunlight, and passed through a 5-mm screen. The used soil was classified as loam-textured soil, which was characterized as follows: soil pH was 7.02 ± 0.47, the soil organic matter was 6.43 ± 0.37 g·kg^−1^, the total phosphorus (TP) content was 0.47 ± 0.012 g·kg^−1^, the available phosphorus (AP) content was 14.75 ± 1.36 mg·kg^−1^, the available nitrogen (AN) content was 32.41 ± 6.12 mg·kg^−1^, and the available potassium (AK) content was 123.46 ± 15.02 mg·kg^−1^.

### Experimental design, treatments, and cultural practices

**Figure 1:**
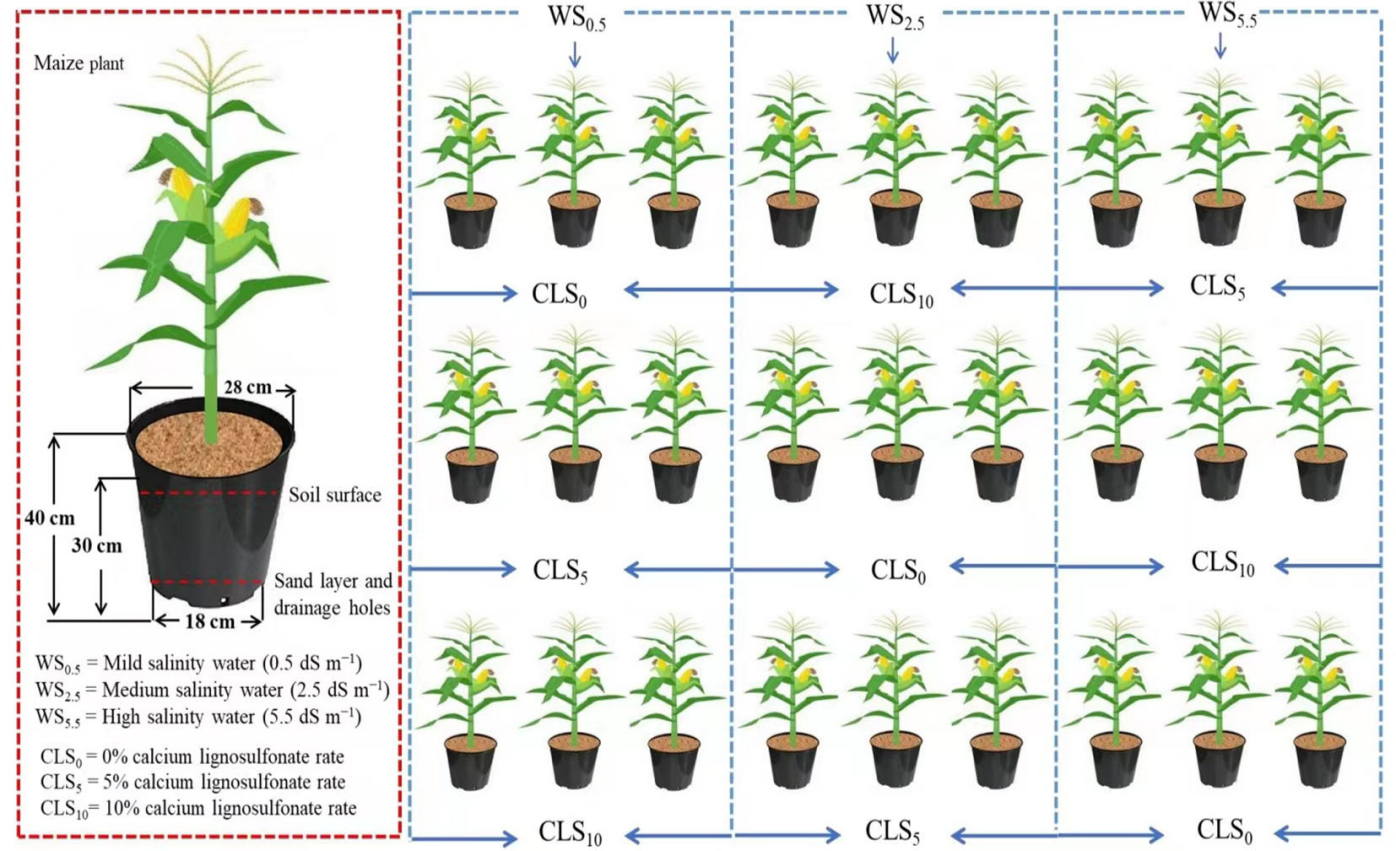
Illustration displaying the experimental pot setup and experimental design.

This study was carried out using a randomized complete block design using three repetitions (**Figure 1**). The first factor was saline water used for irrigation, which imposed three levels with an electrical conductivity (EC) of 0.5 dS/m as control, 2.5, and 5.5, representing WS_0.5_, WS_2.5_, and WS_5_, respectively. The addition made the saline irrigation of 50% sodium chloride (NaCl) and 50% calcium chloride (CaCl_2_) by weight in well water. NaCl was supplied to water used for irrigation to attain the proposed irrigation water salinity levels (WS2.5 and WS5.5) (melting 0.5843 g of NaCl in 1 liter of water increased the EC by 1 dS/m).

The second factor was calcium lignosulfonate (CLS) in addition to three levels: CLS_0_ (0%), CLS_5_ (5%), and CLS_10_ (10%) on a weight basis. A group of 27 PVC vessels were installed in an uncontrolled shelter roofed with elastic film. Every experimental vessel (depth: 40 cm, diameter: 25 cm) had tiny holes at the bottom and was occupied with 10 kg dry soil (**Figure 1**).

Later, CLS amounts were supplied and homogenized with the soil. The quantity of CLS at 5% (CLS_5_) was calculated on a weight basis, supposing the soil bulk density of 1 g/cm^3^. The quantity of CLS at the level of 10% (CLS_10_) enlarged twice to accomplish the rate of 10% (CLS_10_), whereas no CLS was added to the rate of 0% (CLS_0_).

After filling the pots with the soil mixed with the corresponding rates of CLS, the superphosphate (12%, P_2_O_5_), urea (46% N), and potassium chloride (60% K_2_O) as chemical fertilizers were added to the mixture according to the soil test. The pot moisture holding capacity was measured for each rate of CLS. Then, the maize variety, so-called Xianyu 335, was used. The seeds were sown in soil peat moss at three seeds per plug tray cell and thinned to one plant per hole 3 weeks after the germination. Then, the seedlings were transplanted to the pots and irrigated to 100% of pot water-holding capacity every other day for a week. Afterward, irrigation water salinity treatments were implemented every other day, and water amounts were determined based on the water quantity needed to increase the moisture content to 100% of the pot water-holding capacity.

### Soil analysis

For chemical investigation, the soil samples were obtained from the topsoil layer (0 cm–20 cm). A pH meter estimated soil acidity (pH) in soil/water extract (1/2.5). The EC of soil was assessed by measuring the extract through an EC meter. Some samples were placed in a fridge at 4°C to estimate the dissolved organic carbon (DOC). Additional samples were also saved to measure soil easily oxidative carbon (EOC) and soil total organic carbon (TOC). The TOC amount was computed by the titration of ferrous ammonium sulfate and the potassium dichromate oxidation (Lu, 1999). The soil DOC level was computed in the soil/water extract at 25.5°C after shaking for 1 h (Jiang et al., 2006) and rotating at 4,500 r·min^−1^ for 15 min (Bankó et al., 2021). The occasioning supernatant was passed through a 0.45-mm screen, and the attained blend was measured by the titration of ferrous ammonium sulfate and the oxidation of potassium dichromate. The EOC amount was estimated by reacting finely dried samples with 333 mmol·L^−1^ KMnO_4_ through shaking at 60 r·min^−1^ for 60 min (Blair et al., 1995). After that, the resultant was centrifuged for 5 min at 2,000 r·min^−1^. The obtained mixture was diluted, and its absorbance was noted at 565 nm using a spectrophotometer. Subsamples were added to a blend of salicylic acid and selenium sulfate to measure the ion contents of potassium (K^+^), calcium (Ca^++^), and sodium (Na^+^) in the soil. The mixture was transferred to the ingestion cylinders, which were heated for 30 min at 100°C. The temperature was then raised to 380°C for 180 min using a hotplate (YKM-36, Shanghai, China) (Jackson, 2005). Soil K^+^, Ca^++^, and Na^+^ contents were identified by the flame photometer method (FB640N, Wincom, Hunan, China) (Ghosh, 1993).

### Measurements of root growth traits

At physiological maturity (R6), plants were collected, partitioned into grains, stems, and leaves, positioned in the oven for 3 days at 75°C, and balanced to get dry weights. Plant samples for each plant part were milled, sieved using a 1-mm mesh, and kept in the paper bags for further analysis. The soil/root tubes for each treatment were collected, and roots were isolated by carefully washing the soils. The plant roots were dried with paper tissues, and the fresh root mass was determined. The methylene blue dyeing method estimated the root active adsorption area (RAAA) using fresh root samples (Zhang et al., 1994). The root dry weight (RDW) was measured from the fresh root biomass and the water amount of the dried root.

### Measurements of shoot growth and physiological traits

During the vegetative (tassel, VT) and reproductive (dent, R5) growth stages, data regarding plant height (PH, cm), stem diameter (SD, cm), leaf number per plant (NL), and leaf length (LL, cm) were recorded in triplicate. The PH was identified from the soil surface unit at the shoot’s top. The SD was identified through a digital caliper (CD 7303V, China). The NL per plant was counted, and LL was measured using a meter tap.

The photosynthesis level (Pn, µmolCO_2_ m^2^/S) of the leaf was measured through an opened-flow gas exchange apparatus. The Pn rate was measured in three replications of leaves between 08:40 and 10:50 on a sunshiny day through a moveable photosynthesis apparatus (LI-6400XT, LI-COR, USA). The leaf deoxyribonucleic acid (DNA) content was measured at the reproductive (milk, R3) growth stage and detected by flow cytometry analysis. Leaf subsamples were separated into tiny slashes and placed in 50 mM KCl, 5 mM HEPES, 1 mg/mL dithiothreitol (Sigma, St. Louis, MI, USA), 0.2% Triton X-100 (nucleus isolation buffer), and 10 mM MgSO_4_. The samples were passed over a 33-mm net. The nuclei were positioned in paraformaldehyde (4%) for half an hour, sedimented (200 g, 10 min, 4u C), and then suspended in a separation buffer (Sheteiwy et al., 2021a).

### Measurements of the leading enzymes and antioxidant activity in crop

At physiological maturity (R6), the root glutamine synthetase [RGS, μmol/(g·h)] ability was assessed using a standard method by Chen et al. (2021). The nitrate reductase [RNR, µg/(g·h)] activity of the root was assessed by the investigation blend, including 1-g fresh roots sited in 25 mM K_3_PO_4_ buffer, 10 mM KNO_3_, 0.2 mM NADH, 5 mM NaHCO_3_, and 5-μL extract in a final quantity of 0.5 mL. The attained reaction was suspended by delivering 50 μL of 0.5 M Zn(CH_3_COO)_2_. Then, 50 μL of 0.15 mM phenazine methosulphate was delivered to oxidize the excessive NADH. The mixture was centrifuged for 5 min at 10.000× g. The quantity of NO_2−_ was detected by blending 500 μL of supernatant with 250 μL of 1% sulfanilamide obtained in 1.5 N HCl and 250 μL of 0.02% N-(1-naphthyl) ethylene-diamine dihydrochloride. The absorbance was logged at a 540-nm wavelength using the spectrophotometric method (Ogawa et al., 1999).

At the vegetative (tassel, VT) and reproductive (dent, R5) growth stages, subsamples (0.5 g) of the ground flag were frozen in liquid N. They were then placed in a mixture of 15% glycerol, 1 mM EDTA, 0.1% Triton X-100, 1 mL Tris–HCl (pH 7.8), and 14 mM 2-mercaptoethanol. The consequent blend samples were rotated for 12,000× g at 4°C for 10 min. The glutamine synthetase [LGS, µmol/(g·h)] activity of the leaf was identified by identifying glutamyl hydroxamate formation in the mixture at 540 nm after reaction with FeCl_3_ (10%): TCA (24%): HCl (6 mol/L) at 1:1:1 ratio. The leaf nitrate reductase [LNR, µg/(g·h)] activity was identified through a standard method by Baki et al. (2000). Briefly, 0.2 g of the crushed leaf was kept for 30 min at 30°C in a dark place with 0.2 M KNO_3_, 9 mL of medium consisting of 25% isopropanol, and 0.1 M sodium phosphate buffer (acidity 7.2). The spectrophotometer detected the discharged nitrite at a wavelength of 540 nm.

The leaf’s malonaldehyde (MDA) amount was identified by a standard method of Zhou and Leul (1999). Briefly, a flag tissue sample (0.25 g) was added to liquid N by supplying 10% (w/v) TCA (5 ml). The resulting mixture was rotated at 4,000× g for 15 min at 4°C. Later, the supernatant (2 mL) was submitted to 0.67% (w/v) thiobarbituric acid (2 mL), persisted at 100°C for 30 min, and finally transmitted to an ice bath. The subsequent samples were rotated at 4°C at 2,000× g for 5 min. The spectrophotometer recorded the supernatant absorbance at 532-nm, 600-nm, and 450 nm wavelengths. The hydrogen peroxide (H_2_O_2_) level of the supernatant was logged at 390 nm by the spectrophotometer. The leaf’s relative water content (RWC, %) was estimated by drying samples at 75°C for 2 days in the oven, where dry mass was achieved afterward by sealing the targeted leaf for 2 days in deionized water. Then, the leaves were dried before reaching the turgid mass (Sheteiwy et al., 2019).

### Measurements of nutrient crop removal

The wet digestive tubes for the seed, stem, leaf, and root were arranged to measure plant tissues’ K, Ca, and Na nutrient concentrations (%). The digestive tubes were supplied with 0.5 g of each plant part powder. A 1-g selenium reagent mixture was placed in the tube and supplied with 10 mL of concentrated H_2_SO_4_ (Jackson, 2005). The K, Ca, and Na percentages in the tissues of root, stem, leaf, and grain were measured by the flame photometry technique (Tandon, 1993). The amounts of K, Ca, and Na nutrients in crop removal were estimated by the following equations (Mosier and Syers, 2004; Bandaogo et al., 2015):


(1) NAG= (NCG%×GDW/100)

(2) NAL= (NCL%×LDW/100)

(3) NAS= (NCS%×SDW/100)

(4) NAR= (NCR%×RDW/100)

where NAG is the nutrient accretion in grain (mg/plant), NCG is the nutrient concentration in the grain (%), GDW is the grain dry weight (g/plant), NCL is the nutrient concentration in the leaf (%), LDW is the leaf dry weight (g/plant), NAS is the nutrient accretion in the stem (mg/plant), NCS is the nutrient concentration in the stem (%), SDW is the dry weight (g/plant), NAL is the nutrient accretion in leaf (mg/plant), NAR is the nutrient accretion in the root (g/plant), NCR is the nutrient concentration in root tissues (%), and RDW is the root dry weight (g/plant).

### Approval of salt resistance model to corn

As proposed by Maas and Hoffman (1977), the model of salt tolerance was employed in corn yield as a development factor to standardize the level of decline of the factor with increasing salt stress, as indicated by its ECe (excluding the threshold value for assumed salinity and soil amendment). With yield used as a development factor, the salt resistance model (Maas and Hoffman, 1977) is given by the following equation:


(5) 2 YaYM=1−(ECe−ECethreshold)×b100,

where Y_a_ is the average achieved corn yield for a given CLS addition and saline irrigation treatment (g); Y_m_ is the average highest corn production (grain yield) achieved by the CLS_0_ control treatment (g) for the relevant CLS addition; EC_e_ is the soil salinization threshold value (dS m^−1^); ECe is the soil salinization excluding the threshold value (dS m^−1^); and b is the slope value denoting the degree of decreasing yield with rising ECe excluding the threshold value.

### Evaluation of seed embryo ultramorphology, yield, grain chemical structure, and quality

To identify differences in corn embryo ultramorphology for the different treatments, a scanning electron microscope was employed following a standard protocol (Wei et al., 2009). The sonication in dehydrated alcohol dehulled a grain sample (0.1 g) for 5 min. Then, grains were sliced by carpet tape to get cross sections sputter-coated to a thinness of 30 nm with a gold object in a vacuum-coating device. Silver dye was enclosed to the bottom of the seed cuts to avoid the charge on its surface. The seed endosperm ultramorphology engaged to the scanning electron microscope (JEOL JSM-1300) was checked on the core endosperm of a transverse unit at a 15-kV accumulating voltage, and the pictures for each sample were skimmed. The seed chemical structure was identified by calculating the percentages of protein, fat, and starch, according to Pan et al. (2013), using an infrared grain analyzer (FOSS, Hilleroed, Denmark). The seed’s digestive tubes were used where nutrient contents in the grains were determined using the spectrophotometer through the indophenol-blue protocol for N (Novozamsky et al., 1974) and the Barton protocol for P (Jones, 2001). Silicon level (%) was measured using a standard protocol by Zhao et al. (2019).

### Data statistical analysis

The collected data were processed by the IBM-SPSS-19 statistical package, employing the analysis of variance (two-way ANOVA) and performing the process of the general linear model. Once the P values were significant, the obtained mean values were compared at the 0.05 significance level using Duncan’s multiple range test. All collected values are the averages of three replications.

## Results

### Calcium lignosulfonate addition enhanced soil chemical properties under salinity stress

ANOVA results exhibited that under the similar calcium lignosulfonate (CLS) addition rate, increasing the water salinity (WS) stress during WS_0.5_, WS_2.5_, and WS_5.5_ considerably enlarged the soil EC, pH, and Na contents while decreasing EOC, DOC, TOC, K, and Ca contents (**Table 1**). Under similar salinity stress, CLS_0_, CLS_5_, and CLS_10_ led to a large decline in the soil EC, pH, and Na^+^ content and a gradual enlargement in the DOC, EOC, TOC, K^+^, and Ca^++^ levels in the soil. Due to the interactive effect, treatment WS_0.5_ × CLS_10_ exhibited the lowest values of EC (3.85 and 3.00), pH (7.28 and 7.11), and Na^+^ (0.23 and 0.08, g/kg) and the highest EOC (6.30 and 4.23 g/kg), DOC (368.1 and 284.1 mg/kg), TOC (15.64 and 13.58 g/kg), K^+^ (0.167 and 0.139 g/kg), and Ca^++^ (0.39 and 0.19 g/kg) contents of the soil at the layers of 10 cm and 20 cm, respectively. However, treatment WS_5.5_ × CLS_0_ showed the greatest values of EC (15.03 and 14.37), pH (8.56 and 8.02), and Na^+^ (1.01 g/kg and 0.67 g/kg) and the lowest values of EOC (1.82 g/kg and 1.02 g/kg), DOC (172.3 mg/kg and 88.6 mg/kg), TOC (10.67 g/kg and 8.85 g/kg), K^+^ (0.120 g/kg and 0.101 g/kg), and Ca^++^ (0.15 g/kg and 0.08 g/kg) contents of the soil at the depths of 10 and 20 cm, respectively.

**Table 1:** Soil properties for different treatments, and a summary of the analysis of variance on the major impacts of salt-stress and calcium lignosulfonate addition rate on soil chemical properties.

Soil depth	WS level	CLS rate	EC (dS/m)	pH value	EOC (g/kg)	DOC (g/kg)	TOC (g/kg)	K^+^ (g/kg)	Ca^++^ (g/kg)	Na^+^ (g/kg)
0 cm–10 cm	WS_0.5_	CLS_0_	4.92 ± 0.67^Ca^	7.73 ± 0.13^Ca^	2.39 ± 0.25^Ab^	208.5 ± 23^Ab^	11.79 ± 0.9^Ab^	0.129 ± 0.02^Ac^	0.21 ± 0.01^Ab^	0.36 ± 0.03^Ca^
		CLS_5_	4.05 ± 0.54^Ca^	7.40 ± 0.20^Ca^	4.97 ± 0.34^Aa^	301.3 ± 11^Ab^	13.53 ± 0.4^Aa^	0.141 ± 0.01^Ab^	0.29 ± 0.03^Aa^	0.27 ± 0.05^Ca^
		CLS_10_	3.85 ± 0.35^Cb^	7.28 ± 0.33^Cb^	6.30 ± 0.14^Aa^	368.1 ± 14^Aa^	15.64 ± 0.6^Aa^	0.167 ± 0.03^Aa^	0.39 ± 0.06^Aa^	0.23 ± 0.02^Cb^
	WS_2.5_	CLS_0_	5.92 ± 0.23^Bc^	8.13 ± 0.11^Bb^	1.98 ± 0.16^Bc^	187.2 ± 30^Bb^	11.10 ± 0.2^Bb^	0.129 ± 0.04^Bc^	0.18 ± 0.05^Bb^	0.64 ± 0.01^Ba^
		CLS_5_	5.61 ± 0.72^Bb^	8.07 ± 0.15^Bb^	4.16 ± 0.23^Bb^	284.2 ± 14^Ba^	12.43 ± 0.2^Bb^	0.145 ± 0.02^Bb^	0.25 ± 0.02^Bb^	0.51 ± 0.03^Bb^
		CLS_10_	4.92 ± 0.14^Ba^	7.95 ± 0.23^Bb^	5.23 ± 0.42^Ba^	319.3 ± 24^Ba^	15.11 ± 0.2^Ba^	0.159 ± 0.05^Ba^	0.31 ± 0.03^Ba^	0.46 ± 0.05^Bb^
	WS_5.5_	CLS_0_	15.03 ± 0.25^Aa^	8.56 ± 0.44^Aa^	1.82 ± 0.35^Cc^	172.3 ± 12^Cc^	10.67 ± 0.3^Cb^	0.120 ± 0.01^Cb^	0.15 ± 0.04^Cb^	1.01 ± 0.07^Aa^
		CLS_5_	13.78 ± 0.34^Aa^	8.32 ± 0.18^Aa^	2.84 ± 0.62^Cb^	207.4 ± 23^Cb^	12.58 ± 0.2^Ca^	0.130 ± 0.02^Ca^	0.18 ± 0.07^Ca^	0.83 ± 0.02^Ab^
		CLS_10_	11.14 ± 0.62^Ab^	8.20 ± 0.22^Aa^	3.52 ± 0.46^Ca^	257.4 ± 19^Ca^	13.71 ± 0.4^Ca^	0.140 ± 0.01^Ca^	0.25 ± 0.03^Ca^	0.75 ± 0.02^Ab^
10 cm–20 cm	WS_0.5_	CLS_0_	3.75 ± 0.54^Ca^	7.53 ± 0.61^Ca^	1.27 ± 0.37^Ac^	117.3 ± 9^Ab^	10.09 ± 0.1^Ab^	0.109 ± 0.02^Ab^	0.12 ± 0.01^Ab^	0.21 ± 0.04^Ca^
		CLS_5_	3.38 ± 0.92^Ca^	7.33 ± 0.1^Ca^	3.47 ± 0.24^Ab^	213.5 ± 25^Ab^	11.83 ± 0.2^Ab^	0.120 ± 0.02^Ab^	0.14 ± 0.04^Aa^	0.16 ± 0.05^Cb^
		CLS_10_	3.00 ± 0.36^Cb^	7.11 ± 0.19^Cb^	4.23 ± 0.18^Aa^	284.1 ± 32^Aa^	13.58 ± 0.3^Aa^	0.139 ± 0.01^Aa^	0.17 ± 0.02^Aa^	0.08 ± 0.01^Cc^
	WS_2.5_	CLS_0_	11.65 ± 0.29^Ba^	7.80 ± 0.12^Ba^	1.28 ± 0.09^Bc^	106.0 ± 11^Bb^	9.21 ± 0.1^Bb^	0.108 ± 0.03^Bb^	0.19 ± 0.05^Bb^	0.39 ± 0.07^Ba^
		CLS_5_	10.83 ± 0.11^Ba^	7.42 ± 0.25^Ba^	3.06 ± 0.11^Bb^	212.7 ± 16^Ba^	10.05 ± 0.2^Ba^	0.118 ± 0.02^Ba^	0.12 ± 0.01^Bb^	0.34 ± 0.02^Ba^
		CLS_10_	8.84 ± 0.09^Bb^	7.16 ± 0.30^Ba^	4.13 ± 0.15^Ba^	220.5 ± 13^Ba^	11.01 ± 0.1^Ba^	0.119 ± 0.01^Ba^	0.15 ± 0.06^Ba^	0.27 ± 0.01^Bb^
	WS_5.5_	CLS_0_	14.37 ± 0.12^Aa^	8.02 ± 0.11^Aa^	1.02 ± 0.02^Cb^	88.6 ± 7^Cc^	8.85 ± 0.1^Cb^	0.101 ± 0.02^Cb^	0.08 ± 0.03^Cb^	0.67 ± 0.04^Aa^
		CLS_5_	12.37 ± 0.23^Ab^	7.87 ± 0.32^Ab^	2.64 ± 0.01^Ca^	127.3 ± 13^Cb^	9.78 ± 0.2^Ca^	0.109 ± 0.03^Ca^	0.11 ± 0.01^Ca^	0.59 ± 0.02^Aa^
		CLS_10_	10.83 ± 0.18^Ac^	7.45 ± 0.24^Ab^	2.98 ± 0.08^Ca^	179.3 ± 16^Ca^	10.67 ± 0.1^Ca^	0.121 ± 0.02^Ca^	0.13 ± 0.03^Ca^	0.52 ± 0.01^Ab^
ANOVA										
WS			*	***	*	***	*	***	**	*
CLS			*	***	**	***	***	***	*	***
WS × CLS			***	***	*	***	**	*	***	**

WS_0.5_, WS_2.5_, and WS_5.5_ denote water salinization (WS) matching 0.5 dS/m^−1^, 2.5 dS/m^−1^, and 5.5 dS/m^−1^, correspondingly. CLS_0_, CLS_5_, and CLS_10_ represent calcium lignosulfonate (CLS) addition rates of 0%, 5%, and 10%, respectively. EC, electrical conductivity; DOC, dissolved organic carbon; EOC, easily oxidative carbon; TOC, total organic carbon; K, potassium; Ca, calcium; Na, sodium. The obtained mean values are significantly different among CLS addition rates (lowercase) or WS treatments (uppercase) (P ≤ 0.05) once followed by a similar letter, according to analysis of variance tests (ANOVA); ***, **, and * denote significant differences at between treatments P ≤ 0.001, 0.01, and 0.05, correspondingly, ns, not significant. Data are mean ± SE (n = 3).

### Calcium lignosulfonate addition encouraged physiological traits and K concentration in root under salt-stress

At physiological maturity (R6), the RAAA, RDW, RNR, and RGS of roots were reduced by increasing the salinity stress during WS_0.5_, WS_2.5_, and WS5_5.5_ whereas the RAAA, RDW, RNR, and RGS of roots enlarged with increasing CLS addition rate during CLS_0_, CLS_5_, and CLS_10_, respectively. In addition, the highest RAAA (6.54 m^2^/plant), RDW (2.61 g/plant), RNR [14.07 µg/(g·h)], and RGS (3.58 mg/g/FW/h) values in CLS_10_ were detected under WS_0.5_. However, the lowest RAAA (2.38 m^2^/plant), RDW (1.74 g/plant), RNR [5.87 µg/(g·h)], and RGS (1.38 mg/g/FW/h) values in CLS_0_ were detected under WS_5.5_ (**Table 2**).

**Table 2:** Root physiological indicators for different treatments and a summary of the analysis of variance on the major impacts of salt stress and calcium lignosulfonate addition rate on root physiological indicators.

Treatment		Root physiological indicator				Ion concentration in the root		
WS stress	CLS rate	RAAA (m^2^/plant)	RDW (g/plant)	RNR (µg/(g·h))	RGS (μmol/(g·h))	K (%)	Ca (%)	Na (%)
WS_0.5_	CLS_0_	3.38 ± 0.04**^Ac^**	1.64 ± 0.04**^Ab^**	7.72 ± 1.4**^Ac^**	2.32 ± 0.1**^Ac^**	0.41 ± 0.03**^Ac^**	0.19 ± 0.02**^Ac^**	0.19 ± 0.01**^Ca^**
	CLS_5_	4.45 ± 0.03**^Ab^**	2.12 ± 0.02**^Ab^**	10.94 ± 1.2**^Ab^**	2.49 ± 0.2**^Ab^**	0.52 ± 0.06**^Ab^**	0.27 ± 0.02**^Ab^**	0.14 ± 0.01**^Cb^**
	CLS_10_	6.54 ± 0.01**^Aa^**	2.71 ± 0.01**^Aa^**	14.07 ± 1.9**^Aa^**	3.58 ± 0.3**^Aa^**	0.73 ± 0.09**^Aa^**	0.38 ± 0.05**^Aa^**	0.07 ± 0.02**^Cc^**
WS_2.5_	CLS_0_	2.91 ± 0.02**^Bc^**	1.23 ± 0.03**^Bc^**	6.09 ± 1.0**^Bc^**	1.91 ± 0.1**^Bc^**	0.32 ± 0.07**^Bc^**	0.14 ± 0.03**^Bc^**	0.27 ± 0.08**^Ba^**
	CLS_5_	3.86 ± 0.03**^Bb^**	1.49 ± 0.01**^Bb^**	7.89 ± 1.1**^Bb^**	2.26 ± 0.2**^Bb^**	0.41 ± 0.02**^Bb^**	0.20 ± 0.01**^Bb^**	0.21 ± 0.03**^Bb^**
	CLS_10_	5.09 ± 0.02**^Ba^**	1.73 ± 0.02**^Ba^**	9.75 ± 0.92**^Ba^**	2.61 ± 0.2**^Ba^**	0.55 ± 0.05**^Ba^**	0.29 ± 0.03**^Ba^**	0.15 ± 0.02**^Bc^**
WS_5.5_	CLS_0_	1.16 ± 0.01**^Cb^**	1.02 ± 0.02**^Cb^**	3.39 ± 0.7**^Cb^**	0.76 ± 0.06**^Cb^**	0.23 ± 0.01**^Cb^**	0.11 ± 0.04**^Cc^**	0.34 ± 0.04**^Aa^**
	CLS_5_	1.99 ± 0.01**^Cb^**	1.35 ± 0.02**^Ca^**	4.51 ± 0.9**^Cb^**	0.99 ± 0.08**^Cb^**	0.35 ± 0.04**^Cb^**	0.15 ± 0.03**^Cb^**	0.28 ± 0.02**^Ab^**
	CLS_10_	2.38 ± 0.03**^Ca^**	1.44 ± 0.01**^Ca^**	5.87 ± 0.7**^Ca^**	1.38 ± 0.03**^Ca^**	0.40 ± 0.05**^Ca^**	0.21 ± 0.06**^Ca^**	0.20 ± 0.05**^Ab^**
ANOVA								
WS		*	***	***	***	*	*	*
CLS		**	***	***	***	***	***	*
WS × CLS		***	ns	***	*	***	**	***

WS_0.5_, WS_2.5_, and WS_5.5_ denote water salinity (WS) matching 0.5 dS/m^−1^, 2.5 dS/m^−1^, and 5.5 dS/m^−1^, correspondingly. CLS_0_, CLS_5_, and CLS_10_ represent calcium lignosulfonate (CLS) addition rates of 0%, 5%, and 10%, respectively. RAAA, root active adsorption area; RDW, root dry weight; RGS, root glutamine synthetase; RNR, root nitrate reductase; K, potassium; Ca, calcium; Na, sodium. The obtained mean values are significantly different among CLS addition rates (lowercase) or WS treatments (uppercase) (P ≤ 0.05) once followed by a similar letter, according to analysis of variance tests (ANOVA); ***, **, and * denote significant differences at between treatments P ≤ 0.001, 0.01, and 0.05, correspondingly, ns, not significant. Data represent the mean values of three replications. Data are mean ± SE (n = 3).

Significant variations in K, Ca, and Na contents in the root tissues were detected among salinity treatments. Under a similar CLS application rate, the root tissues’ K and Ca contents decreased gradually, whereas Na content increased during WS_0.5_, WS_2.5_, and WS5_5.5_, respectively. Under the similar salinity treatment, K and Ca root contents increased gradually while decreasing Na root content during CLS_0_, CLS_5_, and CLS_10_. The highest K (0.73%) and Ca (0.38%) contents and the lowest Na (0.07%) content in the root tissues were recorded by the treatment WS_5.5_ × CLS_10_. However, the lowest K (0.23%) and Ca (0.11%) contents and the highest Na (0.34%) content in the root tissues were recorded by the treatment WS_0.5_ × CLS_0_ (**Table 2**).

### Calcium lignosulfonate addition encouraged cell cycle progression of the leaf under salinity stress

**Figure 2:**
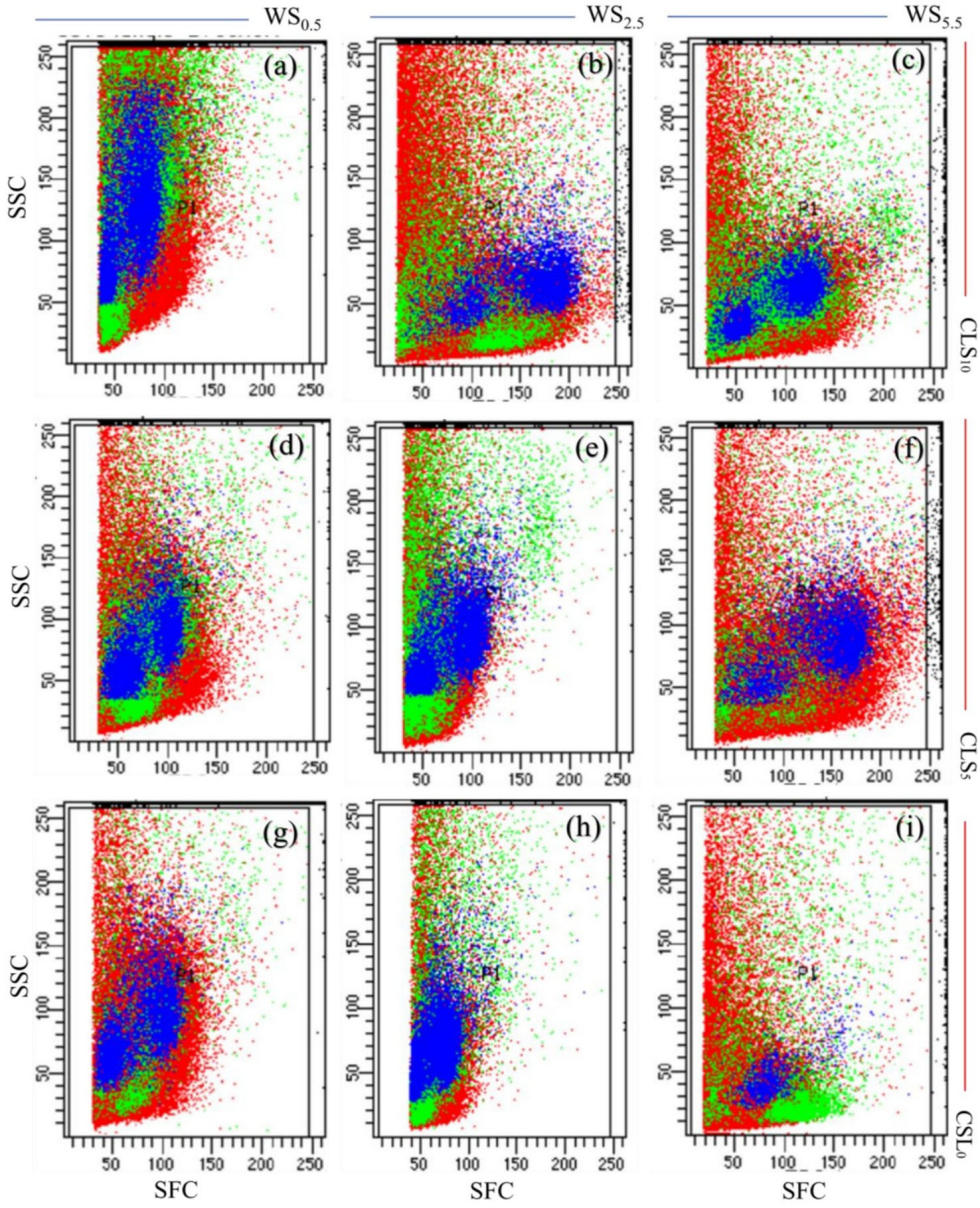
Maize’s leaf cell flow cytometric investigation for different treatments. **(A–C)** represents flow cytometric analysis for plants in soil treated with calcium lignosulfonate (CLS) addition rate of 10% under water salinization (WS) stress of WS0.5, WS2.5, and WS5.5, corresponding to 0.5, 2.5, and 5.5 dS/m, respectively; **(D–F)** represents flow cytometric analysis for plants in soil treated with CLS addition rate of 5% under WS0.5, WS2.5, and WS5.5, respectively; **(G–I)** represents flow cytometric analysis for plants grown in soil treated with CLS addition rate of 0% under WS0.5, WS2.5, and WS5.5, respectively. SSC and FSC donate side scattered and forward scattered.

The results of the flow cytometry investigation presented that CLS addition and salinity stress considerably alerted the cell cycle’s progress at exact stages (**Figures 2A–I**). The findings revealed that each treatment influenced the plant cell progression differently. Maximum values of side-scattered (SSC) light and forward-scattered (FSC) light signals were discovered in maize grown in the CLS_10_ exposed to minor salinity stress under WS_0.5_, signifying superior cell division, size, and granularity (**Figures 2A–C**).

Enhanced values of SSC and FSC were recognized in maize grown in CLS_5_, specifying an enhanced cell division, size, and granularity, with lessening salinity stress during the WS_2.5_ treatments. Thus, the cells’ development was slightly motivated by the variations in the cell’s progress when growing maize in CLS_5_ with decreasing salinity stress during the WS_2.5_ (**Figures 2D–F**). Minimum values of SSC and FSC were noted in maize grown in CLS_10_, demonstrating a negligible developed cell size division and granularity while maximizing salinity stress during the WS_5.5_. Thus, cell progression was noticeably reduced, parallel to changes in cell development when growing maize in CLS_10_ with lightning salinity stress during WS_0.5_ (**Figures 2G–I**).

### Calcium lignosulfonate application enhanced shoot physiological traits under salt stress

**Figure 3:**
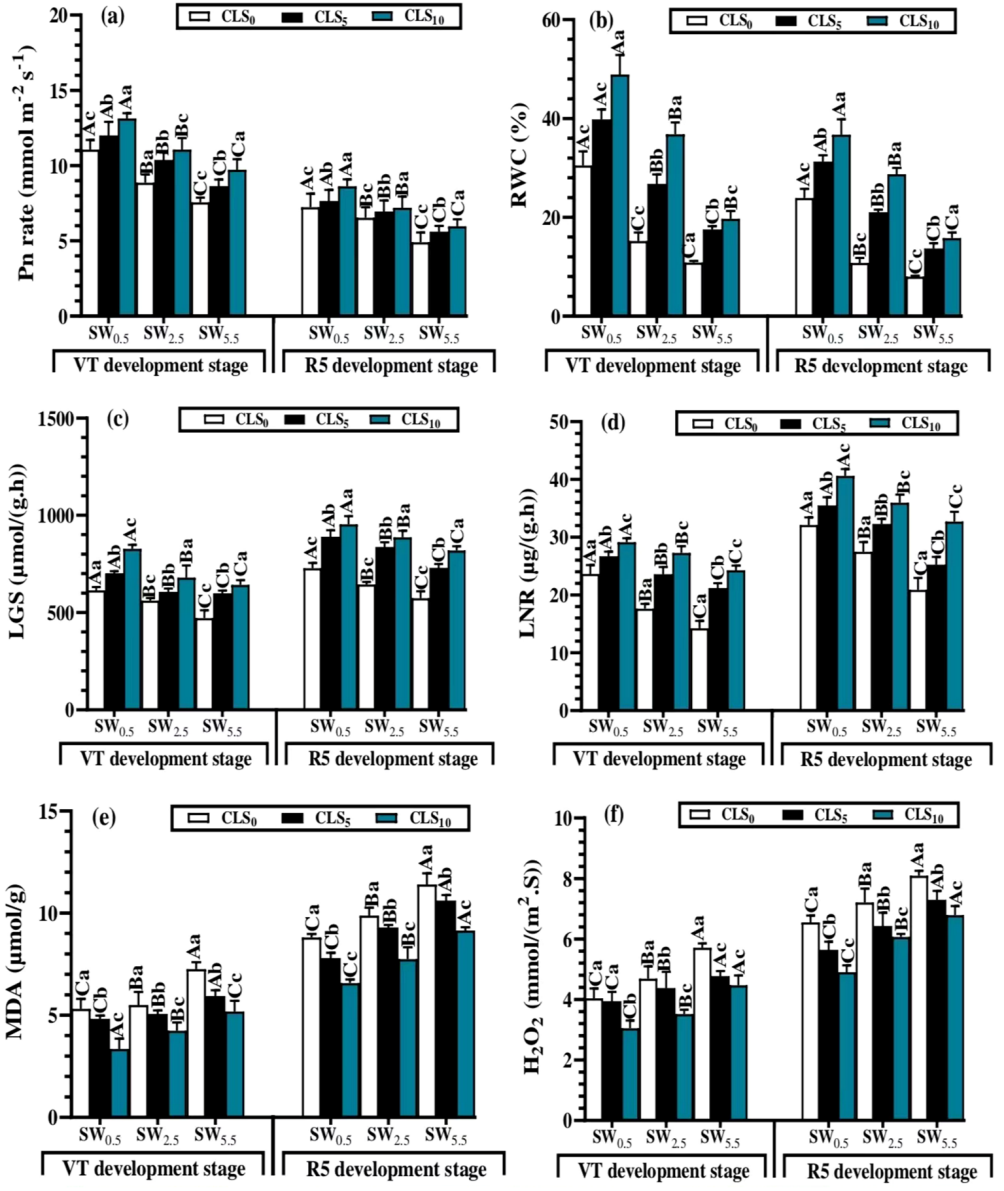
Photosynthesis (Pn) **(A)** and relative water content of leaf (RWC) **(B)**, leaf’s glutamine synthetase (LGS) (c), leaf’s nitrate reductase (LNR) **(D)**, malonaldehyde (MDA) **(E)** and hydrogen peroxide (H2O2) **(F)** mean values as affected by different calcium lignosulfonate addition under salinity stress for different growth stages. WS5.5, WS2.5, and WS0.5 denote water salinization (WS) stress corresponding to 5.5, 2.5, and 0.5 dS/m, respectively. CLS0, CLS5, and CLS10 represent calcium lignosulfonate (CLS) addition rates of 0%, 5%, and 10%, respectively. For the same growth stage, the means are significantly different among CLS addition rates (lowercase) or WS treatments (uppercase) (P ≤ 0.05) once followed by a similar letter, according to the analysis of variance tests. Data are mean ± SE (n = 3). VT represents the tasselling of the vegetative growth stage, and R5 represents the denting of the reproductive growth stage.

During the vegetative (tassel, VT) and reproductive (dent, R5) growth stages, CLS addition significantly enhanced the RWC and Pn in all salinity stress treatments. This impact improved as the amount of CLS added increased. Under the same CLS application level, the salinity stress treatments impacted the RWC and Pn differently, where their values were increased by decreasing salt-stress during the WS_5.5_, WS_2.5,_ and WS_0.5_ treatments. Regarding the interaction of the factors, the greatest Pn (13.13 µmol m^−2^ s^−1^ and 8.63 µmol m^−2^ s^−1^) and RWC (48.93% and 36.73%) were recorded by WS_0.5_ × CLS_0_ at vegetative (tassel, VT) and reproductive (dent, R5) growth stages, respectively. However, the lowest Pn (7.65 µmol m^−2^ s^−1^ and 4.90 µmol m^−2^ s^−1^) and RWC (10.86% and 7.99%) values were observed with WS_5.5_ × CLS_10_ (**Figures 3A, B**).

Given significant differences between different factors, CLS application significantly improved the LGS and LNR under salinity stress treatment. Under the same CLS rate, the salinity stress displayed a varied effect on the LGS and LNR, where their values were improved by reducing salt-stress during the WS_0.5_, WS_2.5_, and WS_5.5_. Given the interaction of the factors, the greatest LGS [827.7 µmol/(g·h) and 953.9 µmol/(g·h)] and LNR [29.12 µmol/(g·h) and 40.59 µg/(g·h)] were recorded by WS_0.5_ × CLS_0_ at the vegetative (tassel, VT) and reproductive (dent, R5) growth stages, respectively. However, the lowest LGS [471.6 µmol/(g·h) and 573.4 µmol/(g·h)] and LNR [14.25 µg/(g·h) and 20.86 µg/(g·h)] were recorded values were observed with WS_5.5_ × CLS_10_ (**Figures 3C, D**).

The MDA and H_2_O_2_ contents decreased significantly with increases in CLS addition amounts, whereas the increasing salinity stress increased MDA and H_2_O_2_ contents. Given the interaction of factors, WS_5.5_ × CLS_0_ resulted in the maximum values of MDA (7.26 µmol/g and 11.41 µmol/g) and H_2_O_2_ [5.71 mmol/(m^2^·S) and 7.11 mmol/(m^2^·S)] contents at the vegetative (tassel, VT) and reproductive (dent, R5) stages, respectively. However, the minimum MDA (3.34 and 6.57 µmol/g) and H_2_O_2_ [3.05 mmol/(m^2^·S) and 4.91 mmol/(m^2^·S)] values were recorded by WS_0.5_ × CLS_10_ (**Figures 3E, F**).

### Calcium lignosulfonate addition stimulated crop growth indicators under salt stress

**Figure 4:**
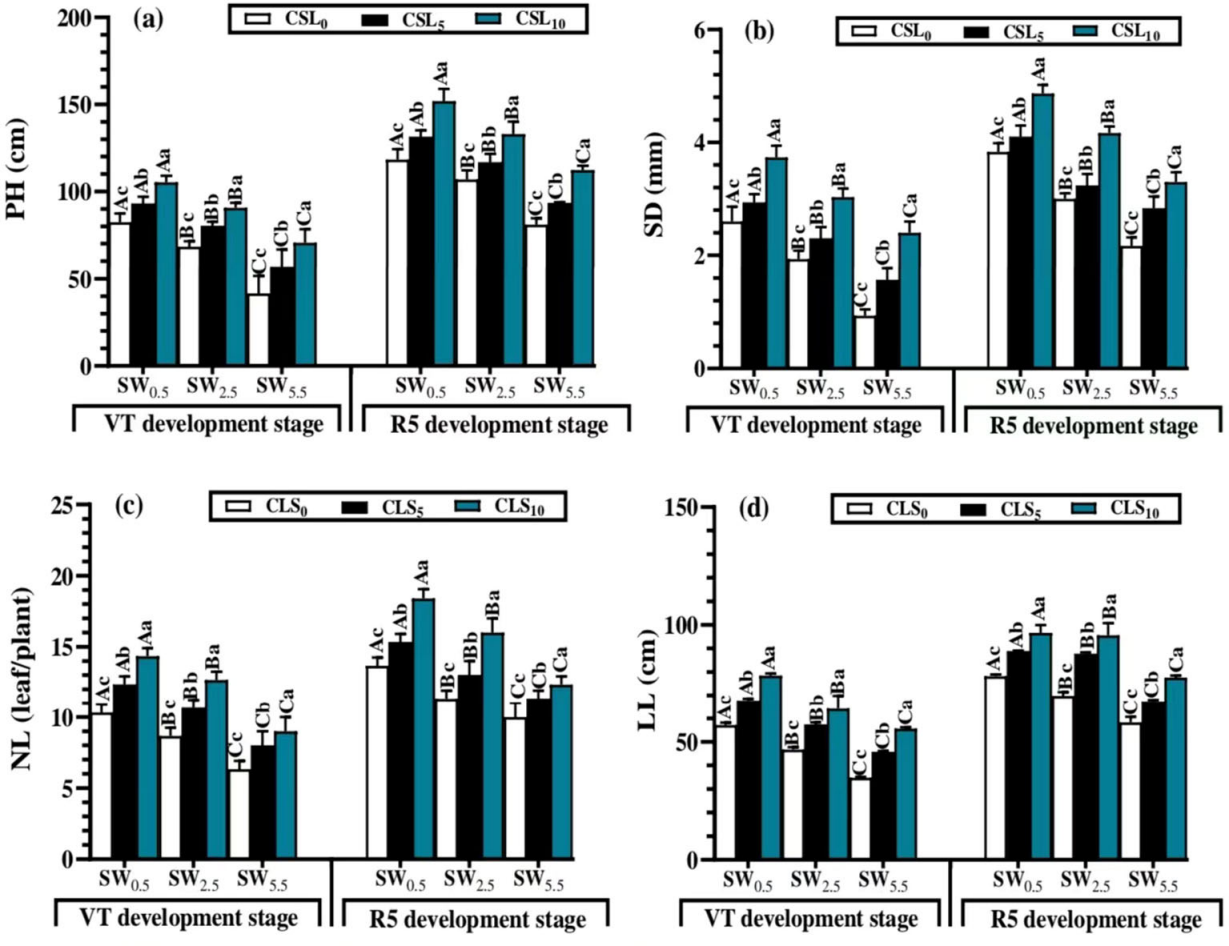
Plant height (PH) **(A)**, stem diameter (SD) **(B)**, number of leaves per plant (NL) **(C)** and leaf’s length (LL) **(D)** mean values as affected by different calcium lignosulfonate additions under salinity stress for different growth stages. WS5.5, WS2.5, and WS0.5 denote water salinization (WS) stress corresponding to 5.5, 2.5, and 0.5 dS/m, respectively. CLS0, CLS5, and CLS10 represent calcium lignosulfonate (CLS) addition rates of 0%, 5%, and 10%, respectively. For the same growth stage, the means are significantly different among CLS addition rates (lowercase) or WS treatments (uppercase) (P ≤ 0.05) once followed by a similar letter, according to the analysis of variance tests. Data are mean ± SE (n = 3). VT represents the tasselling of the vegetative growth stage, and R5 represents the denting of the reproductive growth stage.

As displayed in **Figures 4A–D** at the vegetative growth phase, NL, LL, PH, and SD were unfavorably declined by salinity stress. WS_5.5_ led to lower NL, LL, PH, and SD values under all salt-stress treatments, whereas WS_0.5_ resulted in the greatest values of PH, SD, NL, and LL.

The CLS application showed a substantial augmentation in LL, NL, PH, and SD values under the salt stress treatments. CLS_10_ resulted in greater PH, SD, NL, and LL values under all salt-stress treatments. Regarding interactive effects of the factors, the greatest PH, SD, NL, and LL mean values were recorded by WS_0.5_ × CLS_10_ (105.3 cm, 3.73 mm, 6 leaves/plant, and 78.3 cm), whereas the lower mean values were noticed with WS_5.5_ × CLS_0_ (42 cm, 0.93 mm, 14 leaves/plant, and 34.5 cm).

Similar to the previous phase, salinity stress reduced the growth parameters of maize during WS_0.5_, WS_2.5_, and WS_5.5_. However, CLS addition exhibited a major enhancement in these parameters during CLS_0_, CLS_5_, and CLS_10_, during the vegetative (tassel, VT) and reproductive (dent, R5) growth stages (**Figures 3A–D**). Therefore, the highest PH, SD, NL, and LL values were detected in WS_0.5_ × CLS_10_ (152.5 cm, 4.86 mm, 18 leaves/plant, and 96.6 cm), whereas the smallest values were noted by WS_5.5_ × CLS_0_ (81.2 cm, 2.16 mm, 10 leaves/plant, and 58.3 cm).

### Calcium lignosulfonate addition increased the ion concentrations in maize under salinity stress

The variations in biomass production of corn for various CLS addition rates under salt stress were significant. The biomasses for seeds, leaves, stems, and thus shoot biomass improved with CLS rate increasing during CLS_0_, CLS_5_, and CLS_10_, respectively. Meanwhile, increasing salinity stress considerably reduced the masses of grains, leaves, and stems, and thus shooting. Moreover, the highest biomasses for seeds, leaves, stems, and thus shoot biomass were noticed in WS_0.5_ × CLS_10_ [51.55, 87.06, 164.21 (g/plant)]. However, the lowest values were watched in WS_5.5_ × CLS_0_ [19.31, 42.86, 71.25 (g/plant)] (**Table 3**).

**Table 3:** Nutrient concentration of maize for different treatments, and a summary of the analysis of variance on the major impacts of salt stress and calcium lignosulfonate addition rate on the nutrient concentration (%) of maize.

Treatment		Shoot weight			Ion in leaf			Ion in stem			Ion in grain		
WS stress	CLS rate	Leaf (g)	Stem (g)	Grain (g)	K (%)	Ca (%)	Na (%)	K (%)	Ca (%)	Na (%)	K (%)	Ca (%)	Na (%)
WS_0.5_	CLS_0_	32 ± 1.3^Ac^	60 ± 1.2^Ac^	105 ± 3.2^Ac^	1.2 ± 0.03^Ac^	0.37 ± 0.02^Ca^	0.14 ± 0.02^Ca^	0.26 ± 0.02^Ca^	0.68 ± 0.02^Ac^	0.26 ± 0.05^Ca^	0.57 ± 0.04^Ac^	0.53 ± 0.02^Ca^	0.35 ± 0.08^Ca^
	CLS_5_	44 ± 2.5^Ab^	77 ± 0.5^Ab^	149 ± 4.4^Ab^	1.39 ± 0.04^Ab^	0.43 ± 0.01^Cb^	0.11 ± 0.01^Cb^	0.17 ± 0.01^Cb^	0.73 ± 0.04^Ab^	0.17 ± 0.01^Cb^	0.65 ± 0.01^Ab^	0.59 ± 0.03^Cb^	0.23 ± 0.02^Cb^
	CLS_10_	51 ± 0.9^Aa^	87 ± 1.3^Aa^	164 ± 1.7^Aa^	1.71 ± 0.01^Aa^	0.54 ± 0.02^Cc^	0.06 ± 0.01^Cc^	0.11 ± 0.02^Cc^	0.84 ± 0.04^Aa^	0.11 ± 0.02^Cc^	0.81 ± 0.03^Aa^	0.68 ± 0.06^Cc^	0.17 ± 0.01^Cc^
WS_2.5_	CLS_0_	26 ± 1.1^Bc^	51 ± 0.6^Bc^	83 ± 0.9^Bc^	0.84 ± 0.02^Bc^	0.19 ± 0.01^Ba^	0.18 ± 0.03^Ba^	0.29 ± 0.04^Ba^	0.41 ± 0.06^Bc^	0.29 ± 0.04^Ba^	0.39 ± 0.07^Bc^	0.28 ± 0.06^Ba^	0.46 ± 0.06^Ba^
	CLS_5_	34 ± 1.6^Bb^	64 ± 2.3^Bb^	116 ± 3.1^Bb^	1.13 ± 0.06^Bb^	0.33 ± 0.01^Bb^	0.12 ± 0.08^Bb^	0.2 ± 0.07^Bb^	0.55 ± 0.01^Bb^	0.2 ± 0.08^Bb^	0.47 ± 0.04^Bb^	0.42 ± 0.02^Bb^	0.31 ± 0.07^Bb^
	CLS_10_	43 ± 2.1^Ba^	77 ± 0.7^Ba^	140 ± 5.3^Ba^	1.29 ± 0.02^Ba^	0.39 ± 0.02^Bc^	0.09 ± 0.02^Bc^	0.14 ± 0.03^Bc^	1.65 ± 0.02^Ba^	0.14 ± 0.04^Bc^	0.59 ± 0.05^Ba^	0.51 ± 0.09^Bc^	0.22 ± 0.04^Bc^
WS_5.5_	CLS_0_	19 ± 0.7^Cc^	43 ± 1.0^Cc^	71 ± 0.6^Cc^	0.76 ± 0.05^Cc^	0.11 ± 0.04^Aa^	0.37 ± 0.04^Aa^	0.52 ± 0.06^Aa^	0.37 ± 0.05^Cc^	0.52 ± 0.03^Aa^	0.29 ± 0.03^Cc^	0.22 ± 0.03^Aa^	0.56 ± 0.03^Aa^
	CLS_5_	27 ± 0.5^Cb^	59 ± 0.4^Cb^	99 ± 2.1^Cb^	0.83 ± 0.01^Cb^	0.17 ± 0.03^Ab^	0.25 ± 0.02^Ab^	0.39 ± 0.01^Ab^	0.42 ± 0.03^Cb^	0.39 ± 0.01^Ab^	0.39 ± 0.01^Cb^	0.29 ± 0.01^Ab^	0.39 ± 0.01^Ab^
	CLS_10_	38 ± 1.2^Ca^	65 ± 2.1^Ca^	123 ± 6.3^Ca^	0.96 ± 0.03^Ca^	0.26 ± 0.03^Ac^	0.16 ± 0.02^Ac^	0.27 ± 0.04^Ac^	0.48 ± 0.01^Ca^	0.27 ± 0.02^Ac^	0.49 ± 0.03^Ca^	0.38 ± 0.05^Ac^	0.22 ± 0.02^Ac^
ANOVA													
WS		*	***	**	***	**	**	***	*	**	**	*	***
CLS		***	ns	*	*	**	***	**	***	***	*	**	**
WS× CLS		**	***	*	***	*	ns	*	*	***	**	***	***

WS_0.5_, WS_2.5_, and WS_5.5_ denote water salinity (WS) matching 0.5 dS/m^−1^, 2.5 dS/m^−1^, and 5.5 dS/m^−1^, correspondingly. CLS_0_, CLS_5_, and CLS_10_ represent calcium lignosulfonate (CLS) addition rates of 0%, 5%, and 10%, respectively. Potassium (K), calcium (Ca), and sodium (Na). The obtained mean values are significantly different among CLS addition rates (lowercase) or WS treatments (uppercase) (P ≤ 0.05) once followed by a similar letter, according to analysis of variance tests (ANOVA); ***, **, and * denoted significant differences at between treatments P ≤ 0.001, 0.01, and 0.05, correspondingly, ns, not significant. Data represent the mean values of three replications. Data are mean ± SE (n = 3).

Significant changes (P<0.05) in the ion contents in maize plants’ seeds, stems, and leaves were detected across salinity stress treatments. The maize plants were more reactive with K under WS_0.5_ than WS_5.5_ conditions through the season. The K and Ca percentages in stems, leaves, and grains enlarged when the CLS addition rate increased during CLS_0_, CLS_5_, and CLS_10_, respectively. Due to interaction, the maximum values of K and Ca amounts in leaves (1.71% and 0.54%), stems (1.51% and 0.84%), and grains (0.84% and 0.68%) were obtained under the WS_0.5_ × CLS_10_. However, the lowest K and Ca concentrations in leaves (0.76% and 0.11%), stems (0.52% and 0.37%), and grains (0.29% and 0.22%) were achieved under the WS_5.5_ × CLS_0_ (**Table 3**). Unlike the K and Ca concentrations, the Na percentages in the stems, grains, and leaves of maize were considerably increased by increases in salinity stress and reductions in CLS addition rates. Moreover, the highest Na levels in maize grains, stems, and leaves were obtained under WS_5.5_ × CLS_0_ (0.37%, 0.52%, and 0.56%). However, the lower values were witnessed in WS_0.5_ × CLS_10_ (0.06%, 0.11%, and 0.17%) (**Table 3**).

### Calcium lignosulfonate application enlarged the ions uptake of corn under salinity stress

The findings specified that different CLS addition rates under salinity stress treatments affected Na, K, and Ca accumulation in the leaf, stem, root, and grain. The accumulation of K and Ca in the root, leaf, stem, and grain declined with increasing salinity stress during the WS_0.5_, WS_2.5_, and WS_5.5_, respectively. However, the Na accumulation in the stem, root, grain, and leaf improved during the WS_0.5_, WS_2.5_, and WS_5.5_ (**Table 4**), unlike the K and Ca accumulation trend in root, leaf, stem, and grain, which were realized during CLS_0_, CLS_5_, and CLS_10_. However, the Na uptake in leaf, stem, root, and grain declined with increasing CLS addition rates during CLS_0_, CLS_5_, and CLS_10_, respectively. Due to interaction, the highest K and Ca uptake while the lowest Na uptake in the root (19.1 mg, 10 mg, and 1.83 mg), leaf (881.5 mg, 278 mg, and 30.9 mg), stem (1,314 mg/plant, 731 mg/plant, and 95.8 mg/plant), and grain (1,330.1 mg, 1,117 mg, and 279.2 mg) were recorded by the WS_0.5_ × CLS_10_ treatment. However, the lowest K and Ca uptake while the highest Na uptake in the root (6.7 mg, 1.1 mg, and 3.81 mg), leaf (146 mg/plant, 21 mg/plant, and 71 mg/plant), stem (201 mg, 159 mg, and 230 mg), and grain (206 mg, 157 mg, and 399 mg) were recorded by the WS_5.5_ × CLS_0_ treatment (**Table 4**).

**Table 4:** Nutrient uptake (mg/plant) of corn for various treatments (Equations 1–4) and a summary of the analysis of variance on the major impacts of salt stress and calcium lignosulfonate addition rate on nutrient uptake of maize.

Treatment		Root ion uptake			Leaf ion uptake			Stem ion uptake			Ion uptake in grain		
WS stress	CLS rate	K (mg/plant)	Ca (mg/plant)	Na (mg/plant)	K (mg/plant)	Ca (mg/plant)	Na (mg/plant)	K (mg/plant)	Ca (mg/plant)	Na (mg/plant)	K (mg/plant)	Ca (mg/plant)	Na (mg/plant)
WS_0.5_	CLS_0_	6.7 ± 0.02^Ac^	3.1 ± 0.06^Ca^	3.1 ± 0.09^Ca^	398 ± 8^Ac^	118 ± 5^Ca^	44 ± 1.7^Ca^	607 ± 7^Ac^	409 ± 3^Ca^	156 ± 4^Ca^	601 ± 14^Ac^	559 ± 21^Ca^	369 ± 13^Ca^
	CLS_5_	11.0 ± 0.09^Ab^	5.7 ± 0.03^Cb^	2.9 ± 0.02^Cb^	607 ± 4^Ab^	188 ± 4^Cb^	48 ± 2.5^Cb^	930 ± 11^Ab^	561 ± 11^Cb^	130 ± 11^Cb^	966 ± 7^Ab^	878 ± 8^Cb^	342 ± 10^Cb^
	CLS_10_	19.1 ± 0.05^Aa^	10 ± 0.05^Cc^	1.8 ± 0.05^Cc^	881 ± 2^Aa^	278 ± 6^Cc^	30 ± 1.1^Cc^	1314 ± 35^Aa^	731 ± 15^Cc^	95 ± 6^Cc^	1330 ± 23^Aa^	1117 ± 37^Ca^	279 ± 6^Ca^
WS_2.5_	CLS_0_	3.6 ± 0.2^Bc^	1.7 ± 0.01^Ba^	3.3 ± 0.03^Ba^	216 ± 4^Bc^	49 ± 2^Ba^	46 ± 3.2^Ba^	344 ± 26^Bc^	211 ± 9^Ba^	148 ± 8^Ba^	324 ± 6^Bc^	233 ± 6^Ba^	382 ± 8^Ba^
	CLS_5_	7.7 ± 0.01^Bb^	3.0 ± 0.03^Bb^	3.1 ± 0.01^Bb^	389 ± 6^Bb^	114 ± 2^Bb^	41 ± 5.4^Bb^	594 ± 32^Bb^	352 ± 7^Bb^	127 ± 12^Bb^	544 ± 16^Bb^	487 ± 10^Bb^	359 ± 11^Bb^
	CLS_10_	11.1 ± 0.02^Ba^	5.1 ± 0.04^Bc^	2.5 ± 0.06^Bc^	549 ± 1^Ba^	166 ± 8^Bc^	38 ± 6.5^Bc^	821 ± 54^Ba^	503 ± 19^Bc^	108 ± 3^Bc^	828 ± 22^Ba^	716 ± 23^Bc^	308 ± 5^Bc^
WS_5.5_	CLS_0_	1.9 ± 0.02^Cc^	1.1 ± 0.01^Aa^	3.8 ± 0.04^Aa^	146 ± 3^Cc^	21 ± 1^Aa^	71 ± 3.8^Aa^	201 ± 18^Cc^	159 ± 11^Aa^	230 ± 8^Aa^	206 ± 5^Cc^	157 ± 7^Aa^	399 ± 21^Aa^
	CLS_5_	4.8 ± 0.07^Cb^	2.0 ± 0.02^Ab^	3.4 ± 0.07^Ab^	226 ± 5^Cb^	46 ± 3^Ab^	68 ± 4.1^Ab^	366 ± 26^Cb^	248 ± 8^Aa^	222 ± 11^Aa^	387 ± 14^Cb^	287 ± 11^Ab^	387 ± 7^Ab^
	CLS_10_	7.0 ± 0.05^Ca^	3.2 ± 0.01^Ac^	2.7 ± 0.06^Ac^	368 ± 8^Ca^	100 ± 9^Ac^	61 ± 2.7^Ac^	508 ± 32^Ca^	320 ± 12^Ab^	176 ± 9^Ab^	604 ± 8^Ca^	469 ± 20^Ac^	271 ± 4^Ac^
ANOVA													
WS		***	*	**	**	***	***	***	*	*	**	**	***
SL		***	**	***	***	**	**	*	*	**	**	**	***
WS × SL		*	**	ns	***	***	***	ns	*	**	*	*	***

WS_0.5_, WS_2.5_, and WS_5.5_ denote water salinity (WS) matching 0.5 dS/m^−1^, 2.5 dS/m^−1^, and 5.5 dS/m^−1^, correspondingly. CLS_0_, CLS_5_, and CLS_10_ represent calcium lignosulfonate (CLS) addition rates of 0%, 5%, and 10%, respectively. Potassium (K), calcium (Ca), and sodium (Na). The obtained mean values are significantly different among CLS addition rates (lowercase) or WS treatments (uppercase) (P ≤ 0.05) once followed by a similar letter, according to analysis of variance tests (ANOVA); ***, **, and * denote significant differences at between treatments P ≤ 0.001, 0.01, and 0.05, correspondingly, ns, not significant. Data represent the mean values of three replications. Data are mean ± SE (n = 3).

### Calcium lignosulfonate addition enlarged the maize salt resistance to salinity stress

The salt resistance index for the maize plant is presented as a function of grain yield against the reached ECe mean values for every CLS addition rate (**Table 5**). For CLS_0_, no reduction until 2.09 dS/m of the threshold value was detected in the grain yield. However, surpassing the standard value, corn production declined by 27.78% for each unit enlargement in the salt-stress degree. Regarding the CLS_5_, a substantial rise in the ECe value to 4.02 dS/m was witnessed; however, the slope value was reduced to 14.92%. Moreover, under CLS_10_, a substantial enlargement in the ECe value of 5.96 dS/m was detected; however, the slope value was reduced to 7.34% (**Table 5**).

**Table 5:** Fitted factors of salinity resistance models projected (Equation 5) for various rates of calcium lignosulfonate addition under salinity stress.

Salinity stress	Calcium lignosulfonate rate	b	ECe (ds/m)	R^2^
WS	CLS_0_	27.78**^a^**	2.09**^c^**	0.984
	CLS_5_	14.92**^b^**	4.02**^b^**	0.967
	CLS_10_	7.34**^c^**	5.96**^a^**	0.992

Varied letters in the similar column specify major variances (P ≤ 0.05) under different calcium lignosulfonate addition (SL) rates and water salinity (WS) stress levels. SL_0_, SL_5_, and SL_10_ represent calcium lignosulfonate (CLS) addition rates of 0%, 5%, and 10%, correspondingly; ECe is the soil salinity threshold value (dS m^−1^); b is the slope value.

### Calcium lignosulfonate addition improved the grain quality of maize

**Figure 5:**
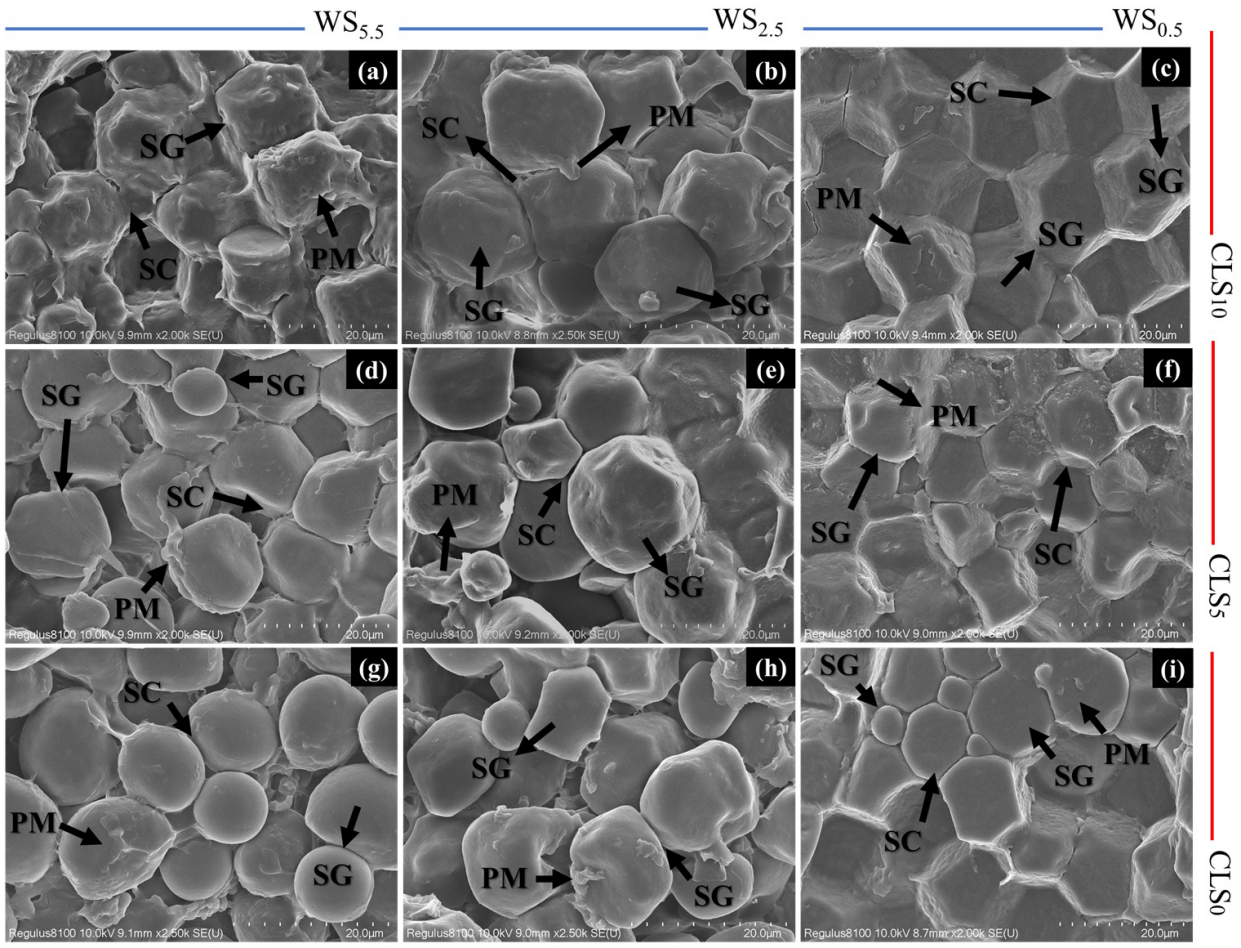
Scanning electron microscopy (SEM) images of the endosperm of maize seeds for different treatments. Starch granules (SG), protein matrix (PM), protein deposits (PD), and compound starch grains (CSG). **(A–C)** represents flow cytometric analysis for plants in soil treated with calcium lignosulfonate (CLS) addition rate of 10% under water salinization (WS) stress of WS5.5, WS2.5, and WS0.5, corresponding to 5.5, 2.5, and 0.5 dS/m, respectively; **(D–F)** represents flow cytometric analysis for plants in soil treated with CLS addition rate of 5% under WS5.5, WS2.5, and WS0.5, respectively; **(G–I)** represents flow cytometric analysis for plants grown in soil treated with CLS addition rate of 0% under WS5.5, WS2.5, and WS0.5, respectively. All images are shown at the same level of magnification, with 20-um scale bars.

The ultramorphology of maize seed embryo altered significantly under different treatments (**Figures 5A–I**). The maize seed embryo exposed to CLS_0_ exhibited many circular starch grains of smaller size and a plethora of unformed starch particles with untidy-edge starch granules. The ultramorphology of maize seed embryo also showed a large increase in the content of protein matrix at the periphery, increasing the injury to the seeds compared with CLS_0_ and WS_0.5_ by increasing salinity stress in the order of WS_0.5_ > WS_2.5_ > WS_5.5_ (**Figures 5A–C**).

However, the ultramorphology of maize grain embryo exposed to CLS_5_ was boosted by enhancements in crystallization and size for starch particles with an obvious drop in protein matrix over reducing salinity stress in the order of WS_5.5_ > WS_2.5_ > WS_0.5_ (**Figures 5D–F**). The positive development in the ultramorphology of maize grain embryos exposed to CLS_10_ developed greater with a greater size for starch grains, well-built starch particles with sharply bounded starch particles. Furthermore, the protein matrix declined, preventing the injury to maize grains compared with CLS_0_ and CLS_10_ by reducing salinity stress in WS_5.5_ > WS_2.5_ > WS_0.5_ (**Figures 5G–I**). Considerable differences in the maize grain’s quality and chemical composition signs were found through different treatments (**Table 6**). Under a similar calcium-sulfonated lignin addition rate, WS_0.5_ boosted proteins, fatty acids, starch, Si, P, and N percentages in the maize grains. During the similar salinity stress, the smallest proportions of protein, fat, starch, Si, P, and N were realized in CLS_0_, followed by CLS_5_, while the greatest percentage was detected in SL_10_.

**Table 6:** Grain quality indicators of corn for various treatments and a summary of the analysis of variance on the major impacts of salinity stress and calcium lignosulfonate addition rate on grain’s quality.

Treatment		Grain chemical composition					
WS stress	CLS rate	Protein (%)	FA (%)	Starch (%)	Si (mg/g)	P (%)	N (%)
WS_0.5_	CLS_0_	6.54 ± 0.22**^Aa^**	3.72 ± 0.42**^Ab^**	68.04 ± 2.3**^Ab^**	1.31 ± 0.02**^Ab^**	0.355 ± 0.02**^Ab^**	1.41 ± 0.01**^Ab^**
	CLS_5_	11.34 ± 0.73**^Aa^**	4.67 ± 0.15**^Aa^**	75.52 ± 6.7**^Ab^**	1.8 ± 0.01**^Ab^**	0.523 ± 0.01**^Aa^**	1.56 ± 0.02**^Aa^**
	CLS_10_	13.65 ± 0.65**^Aa^**	6.81 ± 0.25**^Aa^**	89.29 ± 3.5**^Aa^**	2.35 ± 0.03**^Aa^**	0.703 ± 0.04**^Aa^**	1.95 ± 0.03**^Aa^**
WS_2.5_	CLS_0_	5.87 ± 0.37**^Ba^**	3.09 ± 0.61**^Bb^**	67.15 ± 1.5**^Bb^**	1.00 ± 0.09**^Ba^**	0.282 ± 0.05**^Bb^**	1.18 ± 0.04**^Bb^**
	CLS_5_	7.05 ± 0.32**^Bb^**	4.02 ± 0.43**^Ba^**	71.74 ± 2.6**^Ba^**	1.54 ± 0.04**^Ba^**	0.439 ± 0.01**^Ba^**	1.39 ± 0.01**^Ba^**
	CLS_10_	10.25 ± 0.61**^Bb^**	4.73 ± 0.52**^Ba^**	73.36 ± 3.3**^Ba^**	1.88 ± 0.03**^Ba^**	0.607 ± 0.02**^Ba^**	1.55 ± 0.02**^Ba^**
WS_5.5_	CLS_0_	6.11 ± 0.29**^Cd^**	2.84 ± 0.17**^Cb^**	67.02 ± 4.7**^Cb^**	0.84 ± 0.05**^Cb^**	0.189 ± 0.03**^Cb^**	0.84 ± 0.04**^Cb^**
	CLS_5_	7.04 ± 0.42**^Cc^**	3.67 ± 0.25**^Ca^**	69.82 ± 5.5**^Ca^**	1.01 ± 0.06**^Ca^**	0.312 ± 0.01**^Ca^**	1.13 ± 0.06**^Ca^**
	CLS_10_	8.92 ± 0.46**^Cc^**	4.45 ± 0.32**^Ca^**	73.35 ± 1.8**^Ca^**	1.45 ± 0.03**^Ca^**	0.435 ± 0.01**^Ca^**	1.37 ± 0.03**^Ca^**
ANOVA							
WS		*	*	**	*	*	*
CLS		*	**	***	**	**	*
WS× CLS		**	*	***	ns	***	*

WS_0.5_, WS_2.5_, and WS_5.5_ denote water salinity (WS) corresponding to 0.5 dS/m^−1^, 2.5 dS/m^−1^, and 5.5 dS/m^−1^, respectively. CLS_0_, CLS_5_, and CLS_10_ represent calcium lignosulfonate (CLS) addition rates of 0%, 5%, and 10%, respectively. FA, Si, P, and N indicate fatty acid, silicon, phosphorus, and nitrogen, respectively. The obtained mean values are significantly different among CLS addition rates (lowercase) or WS treatments (uppercase) (P ≤ 0.05) once followed by a similar letter, according to analysis of variance tests (ANOVA); ***, **, and * denote significant differences at between treatments P ≤ 0.001, 0.01, and 0.05, correspondingly, ns, not significant. Data represent the mean values of three replications. Data are mean ± SE (n = 3).

Due to the combination, the WS_0.5_ × CLS_10_ treatment showed the greatest proportions of protein, fat, starch, Si, P, and N in the maize grains. Conversely, the WS_5.5_ × CLS_0_ treatment exhibited the smallest proportions of protein, fat, starch, Si, P, and N in the maize grains.

## Discussion

**Figure 6:**
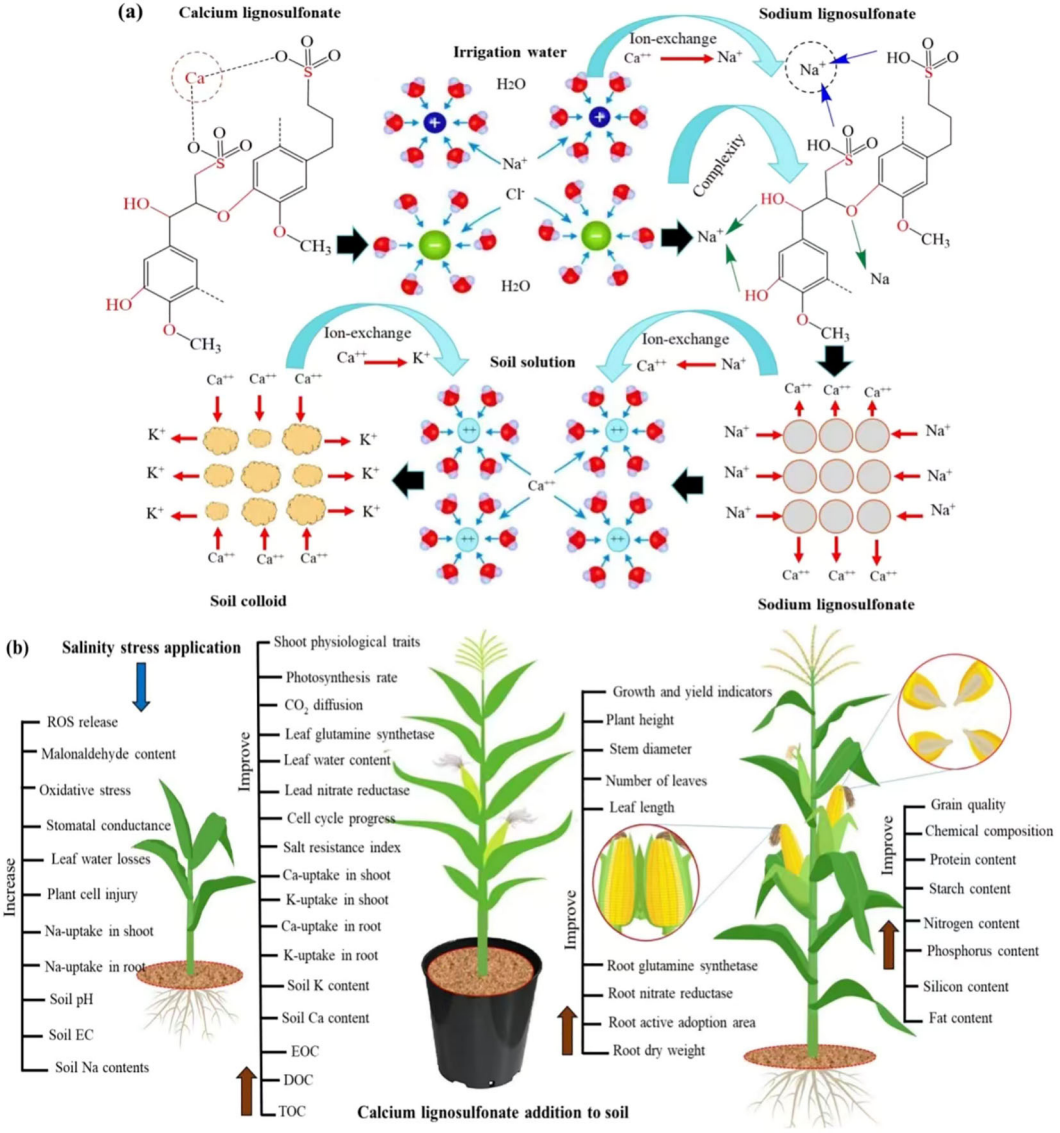
**(A)** Comprehensive model showing the working mechanisms of calcium lignosulfonate (CLS) in salt stress mitigation. **(B)** Comprehensive model presenting the mechanism by which CLS application improves physiological traits and grain quality of maize under salinity stress. K^+^, potassium ion; Ca^++^, calcium ion; Na^+^, sodium ion, and Cl^-^, chloride ion; EOC, easily oxidative carbon; DOC, dissolved oxidative carbon; and TOC total organic carbon.

This study indicates that the maize plant obviously displayed much resistance to salinity stress levels when soil was amended with calcium lignosulfonate (CLS), as revealed by increased root and shoot lengths, grain yield, and higher protein, starch, and fat contents of grain as well as lower cell damage due to lower reactive oxygen species production. This study also reveals that CLS addition could improve the physiological traits, nutrient uptake, and grain quality of maize and soil chemical characteristics under salinity stress. Accumulation of sodium (Na+) in soil markedly causes ion imbalance and toxicity to plants by limiting the competitive absorption of nutrients such as potassium Ca^++^ and K^+^ (Shen et al., 2016). Two potential working mechanisms could be attributed to Na^+^ mitigation using the CLS application. The first and maim mechanism is attributed to Na^+^ adsorption by amble hydrophilic carboxyl and hydroxyl groups on the lignin surface by complexing (Liu et al., 2020). The second mechanism is Na^+^ adsorption by ion exchange with the Ca^++^ existing on the CLS surface (**Figure 6A**). Therefore, the reduction in soil pH and EC with increasing CLS application was due to the soluble nutrients, in particular Ca^++^ and K^+^, entering the soil solution (**Table 1**). Moreover, the increases in EOC, DOC, and TOC with increasing CLS application ascertains that the CLS degeneration improved the quantities of released Ca^++^ and K^+^ in the soil. This finding is in line with the achieved results of an earlier investigation (Elsawy et al., 2022). The reduction in EOC, DOC, and TOC under salt stress specifies that high salt stress decreased the CLS degradation, thus lessening the level of released K^+^ and Ca^++^ in soil solution (Elsawy et al., 2022). Moreover, increasing the salinity stress level increased Na^+^ within the soil, thus increasing the soil pH. However, increasing CLS application decreased Na^+^ in the soil, thus alleviating the undesirable influence of salinity stress on the maize crop.

Root physiology was enhanced under WS_0.5_ × CLS_10_ by generating heavier roots connecting to the soil through superior RAAA, indicating abundant K^+^ and Ca^++^ to roots. Conversely, the deficiency of K^+^ and Ca^++^ with salinity stress under WS_5.5_ × CLS_0_ could limit the root’s growth and, thus, RAAA. Equally, the maize root growth physiology relied on the variations in nutrient obtainability in soil (Vetterlein et al., 2022; Sadak and Dawood, 2023). Also, many crops exhibited considerable tolerance to salinity stress when soil amended with CLS, as shown by increased root growth traits (Kok et al., 2021; Elsawy et al., 2022).

A larger RNR and RGS identify sufficient root-nutrient intake compared with a smaller RNR and RGS (Li et al., 2021a). Therefore, the high RNR and RGS under WS_0.5_ × CLS_10_ show an improved K and Ca nutrient availability while reducing the accumulation of Na in the root. In contrast, the low RNR and RGS define decreased K and Ca nutrient availability and increased accumulation of Na in the root, thus reducing root activity under WS_5.5_ × CLS_0_. In this study, increasing the CLS addition rate under salinity stress increased the soil’s ability to fix Na^+^ ions while increasing the released K^+^ and Ca^++^ ions, letting the plant’s root maximize K and Ca uptake while minimizing Na uptake. Consistently, enzymatic activity and K and Ca uptake of maize roots in the CLS-treated soil were higher than in non-treated soil (Elsawy et al., 2022).

The flow cytometry results under the WS_5.5_ treatment are ascribed to the reduction of plant growth under extreme salinity stress, which reduces plant cell size and progression due to limited cell division (Sheteiwy et al., 2021b). The improvements in rice development regarding cell division and size under WS_0.5_ are due to minor salinity stress, which encourages plant cell size, progression, and division (Sheteiwy et al., 2019). The leaf cells’ progression, size, and division under CLS_10_ were significantly motivated compared with the cells’ progress, size, and division under CLS_0_. This enhancement in the cells’ progress, size, and division is due to the optimal growth conditions under CLS_10_ treatment.

The decrease in Pn and RWC of maize grown under WS_5.5_ × CLS_0_ could result from stomatal conductance started by the larger impact on the guard cells under high salinity stress. Thus, the reduction in CO_2_ diffusion and the RWC under WS_5.5_ × CLS_0_ conditions was also caused by the severe drop in the photosynthetic process due to the over-creation of radical oxidative species (ROS) in particular H_2_O_2_ formation. Consistently, it has been reported that crops must modify to these extreme conditions by eliminating ROS and upregulating enzymes of antioxidation (Hasanuzzaman et al., 2020; Sadak et al., 2022). In addition, Ca^++^ ions could enhance the photosynthesis process by regulating stomata opening and closure (Schulze et al., 2021). Conversely, under WS_0.5_ × SL_10_, the Pn and RWC of the leaf are regulated by increasing the CO_2_ assimilation and the RWC due to a minor impact on the guard cells under minor salinity stress.

The findings of the LNR and LGS enzymes are ascribed to the available nutrients under minor salinity stress of WS_0.5_ × CLS_10_, inhibiting the damage to plant cells by boosting the scavenging sequences, decreasing H_2_O_2_ formation, and minimizing the MDA production. However, the deficiency in available nutrients under high-salinity stress of WS_5.5_ × CLS_0_ motivated the injury to plant cells by disturbing scavenging sequences, boosting the release of H_2_O_2_ formation, and maximizing the MDA production. Consequently, the improvement of the antioxidation defense system led to the mitigation of oxidative cell injury by lessening the H_2_O_2_ and MDA contents in the leaf cell, which is consistent with the results of a pervious study (Abd El-Hameid and Sadak, 2020; Dumanović et al., 2021; Ragaey et al., 2022). Therefore, an improved ROS scavenging system can efficiently alleviate the accretion of H_2_O_2_ and retain a negligible level of ROS for plant development. Hence, CLS encourages the shoot growth of maize over the mitigation of ROS release.

As findings displayed, the PH, SD, NL, and LL were favorably influenced by the improved in the amount of CLS supplied and adversely by the increase in the salinity stress. These results are ascribed to the fact that CLS had a large ability to release Ca, K, and other nutrients, specifically at a high application level by which more nutrients were available to the plant, which was revealed by larger plant development at the different growth stages. These findings are consistent with the findings of (Elsawy et al., 2022), who indicated large enhancements in barley growth and productivity with CLS application. In contrast, the increase in salinity stress could unfavorably affect PH, SD, NL, and LL, certainly in the high salinity stress level. Therefore, all indicators of shoot physiology, including PH, SD, NL, and LL, were lower under WS_5.5_ than those under WS_2.5_ and WS_0.5_ salinity stress levels throughout the different growth stages.

Adding calcium lignosulfonate improved the physiology of root and shoot, demonstrating a favorable impact on the uptake of Ca and K while reducing Na accumulation in maize under salt-stress conditions. These findings are because CLS application could mitigate salt stress in maize by its high transient Na cation binding owing to large acquisition aptitude, so lowering osmotic stress through discharging K and Ca into the soil solution. Recent studies have shown positive responses of crops to CLS application under conditions of low soil quality and plant-nutrient lack (Savy and Cozzolino, 2022; Kang et al., 2024).

Our findings indicated that the seed yield of corn was reduced under high salinity stress. The reduced intake of nutrients, ion toxicity, and ion balance disorder were the fundamental reasons for corn production deterioration under salt stress (El-Bassiouny et al., 2020; Balasubramaniam et al., 2023). However, the CLS addition improved the grain yield of maize under salt stress (Elsawy et al., 2022; Savy and Cozzolino, 2022). Therefore, our findings are ascribed to the capability of CLS to enhance the discharge of K and Ca nutrients into the soil solution while reducing the availability of Na ions.

In the present study, correlations between salinity stress and CLS applied and grain yield of maize were detected. Due to the salinity resistance model, the grain yield of maize was sensitive to salt stress under the non-addition of CLS as the grain yield of maize was reduced by 27.78% per unit elevation in salts above the threshold value (2.09 dS m^-1^). Equally, barley plants presented much tolerance to salinity stress levels when treated with CLS, leading to a larger grain yield (Elsawy et al., 2022). In opposition, after increasing the CLS rate from 5% to 10%, the values elevated from 4.02 dS m^−1^ to 5.96 dS m^−1^; meanwhile, the grain yield of maize reduced by 14.92% and 7.34%. An elevation in the ECe level with the addition of CLS at 10% level could result in a slight decrease in grain yield of maize excess of the ECe threshold standards. Thus, the grain production of maize was further resistant to salinity stress under the addition of CLS at a 10% level.

The ultrastructure of grain embryos attained by SEM confirms the mitigation of injury in grains under WS_0.5_ × CLS_10_ due to the well-organized load of protein particles surrounded by well-formed starch grains in endosperm with the nonappearance of air between starch particles. However, the injury in grain under WS_5.5_ × CLS_0_ is accredited to the incompetent load of the protein bodies with many irregular starch grains, with a considerable decrease in the number of protein particles, therefore increasing the existence of protein matrix with small dimensions and uneven-edge starch grains.

The WS_0.5_ × CLS_10_ treatment could enhance the maize grain composition, essentially affecting the seed’s nutrition. Higher P and N accumulation in grains was due to maize’s efficient growth and mineral nutrition under WS_0.5_ × CLS_10_. The good acquisition of P and N is vital to increasing the corn quality (Razaq et al., 2017; Yuan et al., 2023). As P and N occur in many essential compounds, a slight lack could reduce crop growth and limit P and N accumulation in grain (Sánchez-Rodríguez et al., 2021; Wei et al., 2024). Hence, the sharp decline in the P and N uptake in grains in the WS_5.5_ × CLS_0_ was due to inefficient mineral nutrition of maize under high salinity stress. Thus, the larger the P and N acquisition, the better the nutritional facts of maize seeds, which is consistent with the results of a previous study (Zhang et al., 2023).

Furthermore, P and N are essential elements of amino acids, enzymes, and proteins. Moreover, P and N uptakes enhance maize grain quality through larger carbohydrates, fatty acids, and protein synthesis (Sánchez-Rodríguez et al., 2021; Yuan et al., 2023). Thus, WS_0.5_ × CLS_10_ treatment improved the amino acids, protein, fatty acids, and carbohydrate contents, meanwhile encouraging the silicon contents of grains that improve the filling of proteasome and amyloplast in endosperm with the presence of air among them. Equally, applying CLS on barley contributed to alleviating the salinity stress, leading to higher grain protein content (Elsawy et al., 2022). Moreover, the enhanced P and N uptake by plants improved the amino acids, fatty acids, carbohydrate, and protein proportions and increased the seed’s silicon percentage, thus improving the nutritious facts and seed quality of maize (Sánchez-Rodríguez et al., 2021; Yuan et al., 2023). The lack of P and N uptake under the WS_5.5_ × CLS_0_ enhanced the biotic process of seed filling, thereby the chemical structure and seed quality of maize, thus discouraging the synthesis of amino acids, fatty acids, carbohydrates, and protein and the deposition of seed’s silicon percentage. To support the results of this study, the mechanism by which CLS application improves physiological traits and grain quality of maize under salinity stress (**Figure 6B**). However, long-term experiments should be carried out to define the effect of CLS application on the physical and biological properties of soil. Moreover, open-field trials are required to ascertain the real field response and the degree of the factor’s impact on the chemical properties of soil, development, grain yield, and quality of maize.

## Conclusions

The calcium lignosulfonate (CLS) application could alleviate the salinity stress by enhancing the soil chemical properties, thereby enhancing the K^+^ and Ca^++^ ions entering the soil solution, particularly at a high level of 10%. Meanwhile, the maize root’s growth traits were motivated, achieving a high intake of K^+^ and Ca^++^ ions. Also, the shoot’s physiological attributes were motivated since leaf water losses could be decreased, therefore enhancing the photosynthesis process. Moreover, the plant cell progression cycle was improved while the oxidative stress was minimized as the antioxidant enzymes could achieve the reduction of reactive oxygen species released into the plant cell. Moreover, sufficient K and Ca nutrient uptake in plant parts increased maize tolerance to salinity stress since increasing salinity stress above the threshold values led to a minor decrease in grain yield of maize with the CLS application. Furthermore, the robust glutamine synthetase and nitrate reductase activities could increase protein and starch synthesis during grain filling and were essential to enhancing the protein, fat, and starch contents and increasing the accumulation of mineral nutrients in grains. In opposition, the maize physiology was sensitive to increasing salinity stress without CLS addition. Thus, it led to a sharp reduction in the overall plant growth, K and Ca nutrient uptake, and grain quality, as the antioxidant system does not provide substantial protection against the lipid peroxidation under salinity stress. The present study proposes that CLS application can be employed as a practical approach to enhance soil chemical properties, growth, yield, and grain quality of maize under salinity stress. The present study also provides a theoretical base and practical support for precision water and soil management for sustainable maize production. However, long-term and open-field experiments needed to be conducted to determine the impact of CLS addition on soil physical and biological properties and to confirm the actual field response, and the degree of the factor’s effect on soil properties, growth, yield and grain quality of maize.

## Data Availability

The raw data supporting the conclusions of this article will be made available by the authors, without undue reservation.
